# Simultaneous inference procedures for the comparison of multiple characteristics of two survival functions

**DOI:** 10.1177/09622802241231497

**Published:** 2024-03-11

**Authors:** Robin Ristl, Heiko Götte, Armin Schüler, Martin Posch, Franz König

**Affiliations:** 1 Medical University of Vienna, Center for Medical Data Science, Institute of Medical Statistics, Austria; 2 Merck Healthcare KGaA, Germany; 3 MorphoSys AG, Germany

**Keywords:** Survival analysis, non-proportional hazards, simultaneous inference, multiple testing, randomized clinical trial

## Abstract

Survival time is the primary endpoint of many randomized controlled trials, and a treatment effect is typically quantified by the hazard ratio under the assumption of proportional hazards. Awareness is increasing that in many settings this assumption is a priori violated, for example, due to delayed onset of drug effect. In these cases, interpretation of the hazard ratio estimate is ambiguous and statistical inference for alternative parameters to quantify a treatment effect is warranted. We consider differences or ratios of milestone survival probabilities or quantiles, differences in restricted mean survival times, and an average hazard ratio to be of interest. Typically, more than one such parameter needs to be reported to assess possible treatment benefits, and in confirmatory trials, the according inferential procedures need to be adjusted for multiplicity. A simple Bonferroni adjustment may be too conservative because the different parameters of interest typically show considerable correlation. Hence simultaneous inference procedures that take into account the correlation are warranted. By using the counting process representation of the mentioned parameters, we show that their estimates are asymptotically multivariate normal and we provide an estimate for their covariance matrix. We propose according to the parametric multiple testing procedures and simultaneous confidence intervals. Also, the logrank test may be included in the framework. Finite sample type I error rate and power are studied by simulation. The methods are illustrated with an example from oncology. A software implementation is provided in the R package nph.

## 1. Introduction

The aim of survival analysis in a randomized clinical trial typically is to show a benefit of an experimental treatment over a control treatment. Under the frequent model assumption of proportional hazards, the relative treatment benefit can be quantified with a single parameter, the hazard ratio, which describes the shift of the survival function under treatment compared to the survival function under control at every time point.

When aiming to establish the superiority of a new treatment over control in terms of survival in a randomized controlled clinical trial, the null hypothesis of equal survival functions is typically tested by a logrank test, or an equivalent test based on the Cox model, and a hazard ratio estimate is reported to quantify the treatment effect. Under the assumption of proportional hazards, this approach is efficient in terms of power and allows for unambiguous conclusions on superiority, because one single parameter, the hazard ratio, is sufficient to describe the shift between survival functions at every time point.

Recently, the assumption of proportional hazards has been found to be inadequate or at least questionable in relevant clinical settings.^1–5^ In particular, a delayed onset of treatment effect in immuno-oncology drugs has been discussed as an important source of non-proportional hazards and, moreover, as a setting in which the traditional logrank test loses power and the effect quantification using the hazard ratio as a single summary measure may be flawed. Other sources of non-proportional hazards that have been discussed in the literature are, for example, a heterogeneous patient population with population subgroups that respond differently to treatment, such as long-term survivors, modified efficacy after disease progression, or the need for rescue medication or treatment switching.^5–7^

Several testing procedures have been proposed to remedy the potential loss of power of the logrank test in these settings. In particular, the use of weighted logrank tests has been proposed, putting more weight on event times with a more pronounced anticipated effect.^8,9^ If the pattern of non-proportional hazards is not well known beforehand, a maximum combination (MaxCombo) test of differently weighted logrank tests has been shown to be a robustly powerful method.^5,10–14^ However, in the absence of further assumptions, a significant result for these tests, at first, only implies that there exists a time point for which the hazard function under treatment is less than the hazard function under control. Whether this result translates into a relevant (or in fact into any^9^) benefit in terms of the survival functions needs to be assessed by estimates for parameters that appropriately quantify the differences between survival functions.^6,15^

The interpretation of the parameter estimate of the Cox model can be challenging under non-proportional hazards. In particular, the limiting value of the Cox model hazard ratio estimate will depend not only on the true hazard functions, but also on study design parameters such as recruitment rate, study duration and censoring distribution that affect the timing of observed events. Alternative effect measures, proposed to be used under non-proportional hazards, include the difference in restricted mean survival times,^16,17^ average hazard ratios defined via predefined weighting functions,^18–20^ differences x-year milestone survival probabilities or differences in quantiles of the survival distribution. Several authors have argued that a single such parameter may not be sufficient and differences in survival curves should instead be assessed by a set of summary statistics.^4,6,21^

In this paper, we propose a simultaneous inference framework for a set of multiple parameters, which may include differences in survival probabilities, differences in log survival probabilities, differences in complementary log–log (cloglog)-transformed survival probabilities, differences in quantiles of the survival functions, differences in log-transformed quantiles, an average hazard ratio and the difference in restricted mean survival times. The logrank test, albeit being a non-parametric test, may be included, too. For completeness, the Cox model hazard ratio may be included under the assumption of proportional hazards.

We present multiple testing procedures and simultaneous confidence intervals for pre-specified sets of parameters, such that the family-wise type I error rate is controlled and multiple parameter estimates can be interpreted simultaneously in a confirmatory manner. The multiple testing adjustment is based on the counting process representation of survival function estimates, by which we show that the considered estimates are asymptotically multivariate normal and by which we derive an estimate of their asymptotic covariance matrix. The resulting procedures are more powerful than more simple procedures based on the Bonferroni correction because the latter does not take into account the correlation between estimates.

The paper is structured as follows. In Section 2, we propose a general framework for the multivariate normal approximation of multiple survival parameters and show the application to particular estimates. In Section 3, we describe multiple testing procedures and simultaneous confidence intervals, In Section 4, we perform a simulation study to assess the operating characteristics of the proposed methods in terms of type I error rate and power under different scenarios. In Section 5, we illustrate the proposed methods in a worked example based on survival curves reported by Burtness et al.^22^ (KEYNOTE-048) for the comparison of pembrolizumab versus cetuximab in treating recurrent or metastatic squamous cell carcinoma. A software implementation of all proposed methods is implemented in the R package nph^5^ (see Section 6). We conclude with a discussion.

## 2. Multivariate normal approximation for multiple survival parameter estimates

In this section, we present a general framework for a multivariate normal approximation and the estimation of the covariance matrix for multiple parameters derived from survival functions. We subsequently apply the framework to different parameter estimates that are commonly used to quantify the difference between two survival curves.

### 2.1. General framework based on martingale representation

We consider a control and a treatment group, indexed i=0,1 , including $n_{i}$ independent subjects with observations on possibly censored survival times. Denote with $D_{i}$ the set of observed event times in group i . We assume that the censoring times are stochastically independent of the true event times.

For further notation purposes, let $S_{i}(t)=P_{i}(T_{i}>t)$ be the survival function in group i , with $T_{i}$ a random event time from the respective group. The corresponding hazard function is $\lambda_{i}(t)=(dS_{i}(t)/dt)(1/S_{i}(t))$ , and the cumulative hazard function is $\Lambda_{i}(t)=\int_{0}^{t}\lambda_{i}(s)ds=-\log\;(S_{i}(t))$ . Further, for a given data sample, let $N_{i}(t)$ be the number of observed events in the time interval [0,t] and $Y_{i}(t)$ the number at risk at time t in group i . We denote by ${\hat{\Lambda }}_{i}(t)=\sum_{s\in D_{i},s\leq t}dN_{i}(s)/Y_{i}(s)$ the Nelson–Aalen estimator for the cumulative hazard, by ${\hat{S}}_{i}(t)=\exp\;\{-{\hat{\Lambda }}_{i}(t)\}$ the Nelson–Aalen estimator for $S_{i}(t)$ and by ${\hat{S}}_{i}^{-}(t)=\exp\;\{-\sum_{s\in D_{i},s<t}dN_{i}(s)/Y_{i}(s)\}$ the left continuous version of ${\hat{S}}_{i}(t)$ . Define ${\hat{q}}_{i}(\gamma )=\min\{t:{\hat{S}}_{i}(t)\leq 1-\gamma \}$ as an estimate of the γ -quantile of the survival distribution in group i .

Further we denote by $M_{i}(t)=N_{i}(t)-\int_{0}^{t}Y_{i}(s)d\Lambda_{i}(s)$ the difference between observed and expected events up to time t . $M_{i}(t)$ is a martingale process that is key to the counting process representation of estimates in survival analysis.^8,23^ Finally, we use the wedge operator ∧ to denote the minimum of two quantities.

As a general framework, we aim to quantify the difference in the survival functions under control and treatment by a set of parameters $\theta_{1},\ldots ,\theta_{m}$ . We assume that the true parameters are of the form $\theta_{k}=\theta_{k,1}-\theta_{k,0},k=1,\ldots ,m$ , where $\theta_{k,i}$ is a function of the true survival function and possibly of the true censoring function in group i .

We further assume that the difference between the corresponding estimates ${\hat{\theta }}_{k,i}$ and the true parameter values $\theta_{k,i}$ can be approximated up to an asymptotically negligible residual term by a stochastic integral such that

(1) $${\hat{\theta }}_{k,i}-\theta_{k,i}=a_{k,i}\int_{0}^{t_{k}}H_{k,i}(s)\frac{1}{Y_{i}(s)}dM_{i}(s)+o_{p}(1/\sqrt{n_{i}})$$

where $a_{k,i}$ are constant parameters for which consistent estimates ${\hat{a}}_{k,i}$ exist and $H_{k,i}$ are predictable processes with respect to the martingale process $M_{i}$ for which consistent estimates ${\hat{H}}_{k,i}$ exist. The interval $\left[0,t_{k}\right]$ is the time interval within which data is used to calculate ${\hat{\theta }}_{k,i}$ .

We also assume that ${\hat{\theta }}_{k,0}$ and ${\hat{\theta }}_{k^{\prime },1}$ are asymptotically uncorrelated for all k=1,…,m and $k^{\prime }=1,\ldots ,m$ .

Finally, we assume the asymptotic stability condition holds^24^: for both i∈{0,1} , there exists a function $\rho_{i}$ with values in (0,1) such that for $n_{i}\to \infty$ , $\underset{0<s\leq \max(t_{1},\ldots ,t_{m})}{\sup}|(Y_{i}(s)/n_{i})-\rho_{i}(s)|\to 0$ a.s.

Then, by the multivariate martingale central limit theorem, the vector $\left(\sqrt{n_{i}}({\hat{\theta }}_{1,i}-\theta_{1,i}),\ldots ,\sqrt{n_{i}}({\hat{\theta }}_{m,i}-\theta_{m,i})\right)$ asymptotically follows a multivariate normal distribution with mean zero and variances and covariances given by Fleming and Harrington^8^

$$\begin{array}{r} \text{cov}\left(\sqrt{n_{i}}({\hat{\theta }}_{k,i}-\theta_{k,i}),\sqrt{n_{i}}({\hat{\theta }}_{k^{\prime },i}-\theta_{k^{\prime },i})\right)=a_{k,i}a_{k^{\prime },i}\int_{0}^{t_{k}\wedge t_{k^{\prime }}}H_{k,i}(s)H_{k^{\prime },i}(s)\frac{1}{\rho_{i}(s)}d\Lambda (s)) \end{array}$$

A consistent estimator ${\hat{\Sigma }}_{i}$ for the covariance matrix of $\left({\hat{\theta }}_{1,i},\ldots ,{\hat{\theta }}_{m,i}\right)$ is obtained by replacing $a_{k,i}$ by ${\hat{a}}_{k,i}$ , $H_{k,i}$ by ${\hat{H}}_{k,i}$ , $\rho_{i}(s)$ by $Y_{i}(s)/n_{i}$ and dΛ by $d\hat{\Lambda }=dN_{i}(s)/Y_{i}(s)$ , resulting in

(2) $$\hat{\text{cov}}({\hat{\theta }}_{k,i},{\hat{\theta }}_{k^{\prime },i})={\hat{a}}_{k,i}{\hat{a}}_{k^{\prime },i}\sum_{s\in D_{i},s\leq t_{k}\wedge t_{k^{\prime }}}{\hat{H}}_{k,i}(s){\hat{H}}_{k^{\prime },i}(s)\frac{1}{Y_{i}^{2}(s)}dN_{i}(s)$$

We assume continuous survival distributions such that two events occur at the same time with probability zero. However, in actual applications, event times are not measured precisely but they are typically rounded, for example, to full days, such that tied event times may occur. To account for ties, the term $\left(1/Y_{i}^{2}(s))dN_{i}(s\right)$ in (2) can be replaced by the sum

(3) $$\sum_{j=0}^{dN_{i}(s)-1}\frac{1}{(Y_{i}(s)-j{)}^{2}}$$

if $dN_{i}(s)\geq 1$ , see, for example, Klein and Moeschberger.^25^

Let $\hat{\Sigma_{i}}$ denote the covariance matrix estimate for $\left({\hat{\theta }}_{1,i},\ldots ,{\hat{\theta }}_{m,i}\right)$ with entries according to (2). Then the joint distribution of $\left({\hat{\theta }}_{1},\ldots ,{\hat{\theta }}_{m}\right)$ can be approximated by a multivariate normal distribution with mean $\left(\theta_{1},\ldots ,\theta_{m}\right)$ and covariance matrix $\hat{\Sigma }={\hat{\Sigma }}_{0}+{\hat{\Sigma }}_{1}$ .

### 2.2. Application to specific parameters

To quantify between-group differences, we consider a range of parameters: differences in survival probabilities, differences in log survival probabilities, differences in cloglog-transformed survival probabilities, differences in quantiles of the survival times, differences in log-transformed quantiles, an average hazard ratio and the difference in restricted mean survival times. We also consider the Cox model score test (logrank test) statistic and (under the proportional hazard assumption) the hazard ratio corresponding to the Cox model. For all these parameters, estimators can be constructed that satisfy the assumptions laid out in Section 2.1. Especially, the estimators can be written in the form (1), as will be detailed below. Hence, their joint distribution can be approximated by a multivariate normal distribution, with covariances estimated by equation (2). Based on this approximation, multiple hypotheses tests can be constructed. The specific terms to calculate variance and covariance estimates according to (2) are summarized in Table 1.

**Table 1.** Summary of the expressions required for the variance–covariance estimation according to equation (2) for the considered parameters. The formal definition of the restricted mean survival time estimate ${\hat{\mu }}_{i}$ and the Cox model log hazard ratio estimate $\hat{\beta }$ are given in Section 2.2. In the last column, L is the time-point up to which the (average) hazard ratio, RMST difference or logrank test are calculated.

Parameter of interest $\theta_{k}$	Per group estimate	${\hat{a}}_{k,i}$	${\hat{H}}_{k,i}(s)$	$t_{k}$
Survival difference	${\hat{S}}_{i}(t)$	$-{\hat{S}}_{i}(t)$	1	t
Survival ratio	$\log\;{\hat{S}}_{i}(t)$	−1	1	t
Cumulative-hazard ratio	$\mathrm{cloglog}\;{\hat{S}}_{i}(t)$	$-1/\log\;{\hat{S}}_{i}(t)$	1	t
Quantile difference	${\hat{q}}_{i}(\gamma )$	$\frac{1}{-\hat{\lambda }({\hat{q}}_{i}(\gamma ))}$	1	${\hat{q}}_{i}(\gamma )$
Quantile ratio	$\log\;{\hat{q}}_{i}(\gamma )$	$\frac{1}{-{\hat{q}}_{i}(\gamma )\hat{\lambda }({\hat{q}}_{i}(\gamma ))}$	1	${\hat{q}}_{i}(\gamma )$
Average hazard ratio	$\log\;\int_{0}^{L}{\hat{S}}_{0}^{-}(s){\hat{S}}_{1}^{-}(s)d{\hat{\Lambda }}_{i}(s)$	$\frac{1}{\log\;\int_{0}^{L}{\hat{S}}_{0}^{-}(s){\hat{S}}_{1}^{-}(s)d{\hat{\Lambda }}_{i}(s)}$	${\hat{S}}_{0}^{-}(s){\hat{S}}_{1}^{-}(s)$	L
RMST difference	${\hat{\mu }}_{i}$	1	$\sum_{t_{j}\geq s}\Delta_{t_{j}}{\hat{S}}_{i}(t_{j})$	L
Logrank test	$\int_{0}^{L}\frac{Y_{0}(s)Y_{1}(s)}{Y_{0}(s)+Y_{1}(s)}\frac{1}{Y_{i}(s)}dM_{i}(s)$	1	$\frac{Y_{0}(s)Y_{1}(s)}{Y_{0}(s)+Y_{1}(s)}$	L
Cox model log hazard ratio	$i\hat{\beta }$	$1/\sum_{i=0}^{1}\int_{0}^{L}\frac{Y_{0}(s)Y_{1}(s)\exp\;(\hat{\beta })}{Y_{0}(s)+Y_{1}(s)\exp\;(\hat{\beta })}dN_{i}(s)$	$\frac{Y_{0}(s)Y_{1}(s)\exp\;(i\hat{\beta })}{Y_{0}(s)+Y_{1}(s)\exp\;(\hat{\beta })}$	L

RMST: restricted mean survival time.

#### 2.2.1. Survival probabilities

The estimated difference in survival functions at times t is ${\hat{\theta }}_{j}={\hat{S}}_{1}(t)-{\hat{S}}_{0}(t)$ . The asymptotic properties and the martingale representation for survival function estimates are well established.^8,23^ In particular,

(4) $${\hat{\Lambda }}_{i}(t)-\Lambda_{i}(t)=\int_{0}^{t}\frac{1}{Y_{i}(s)}dM_{i}(s)$$

and by first-order approximation,^8^

(5) $${\hat{S}}_{i}(t)-S_{i}(t)=-S_{i}(t)({\hat{\Lambda }}_{i}(t)-\Lambda_{i}(t))+o_{p}(1/\sqrt{n_{i}})$$

Hence the representation in terms of equation (1) is ${\hat{S}}_{i}(t)-S_{i}(t)\approx -S_{i}(t)\int_{0}^{t}(1/Y_{i}(s))dM_{i}(s)$ .

Alternatively, the ratio in survival probabilities at time t may be of interest, which may be included in the proposed framework on the log-scale in terms of ${\hat{\theta }}_{j}=\log\;{\hat{S}}_{1}(t)-\log\;{\hat{S}}_{0}(t)$ . Since logS(t)=−Λ(t) and by equation (4), the representation in terms of equation (1) is $\log\;{\hat{S}}_{i}(t)-\log\;S_{i}(t)\approx -\int_{0}^{t}(1/Y_{i}(s))dM_{i}(s)$ . Estimates and confidence intervals for the log ratio of survival probabilities may be backtransformed to obtain the respective quantities for the ratio $S_{1}(t)/S_{0}(t)$ at the original scale.

A further common transformation is the cloglog of the estimated survival probability, such that the parameter ${\hat{\theta }}_{j}=\log\;(-\log\;{\hat{S}}_{1}(t))-\log\;(-\log\;{\hat{S}}_{0}(t))$ may be included with the martingale representation $\log\;(-\log\;{\hat{S}}_{i}(t))-\log\;(-\log\;S_{i}(t))\approx -(1/\log\;S(t))\int_{0}^{t}(1/Y_{i}(s))dM_{i}(s)$ . Here, the transformed parameter $\exp\;({\hat{\theta }}_{j})$ may be of interest. This is an estimate for the ratio of cumulative hazards, $\Lambda_{1}(t)/\Lambda_{0}(t)$ (which under proportional hazards corresponds to the hazard ratio).

#### 2.2.2. Quantiles of the survival function

The estimated between-group difference between the γ -quantiles of two survival time distributions is ${\hat{\theta }}_{j}={\hat{q}}_{1}(\gamma )-{\hat{q}}_{0}(\gamma )$ . A first-order approximation is given by ${\hat{q}}_{i}(\gamma )-q_{i}(\gamma )=-(\left({\hat{\Lambda }}_{i}(q_{i}(\gamma ))-\Lambda_{i}(q_{i}(\gamma ))\right)/\left(\lambda_{i}(q_{i}(\gamma ))\right))+o_{p}(1/\sqrt{n_{i}})$ .^26,27^ Again using (4) results in the representation

$$\begin{array}{r} {\hat{q}}_{i}(\gamma )-q_{i}(\gamma )\approx \frac{-1}{{\hat{\lambda }}_{i}(q_{i}(\gamma ))}\int_{0}^{t}\frac{1}{Y_{i}(s)}dM(s) \end{array}$$

Here a consistent estimate ${\hat{\lambda }}_{i}(q_{i}(\gamma ))$ for the hazard at the respective quantile is required. We use a relatively simple approach and estimate ${\hat{\lambda }}_{i}(q_{i}(\gamma ))$ under a locally constant hazard approximation as follows: let $e_{i}$ be the total number of observed events in group i . Define a positive finite constant bandwidth K and the boundaries for a time interval that contains at least $K\sqrt{e_{i}}$ (i.e. for intervals at the boundary) and up to $2K\sqrt{e_{i}}$ (for intervals in the middle of the observed time span) events as $t_{\mathrm{low}}=\max\{0,\max\{t\in D_{i}:N_{i}(t)\leq N_{i}({\hat{q}}_{\gamma ,i})-K\sqrt{e_{i}}\}\}$ and $t_{\mathrm{up}}=\min\{\max\{t\in D_{i}\},\min\{t\in D_{i}:N_{i}(t)\geq N_{i}({\hat{q}}_{\gamma ,i})+K\sqrt{e_{i}})\}\}$ . Here, the maximum and minimum of an empty set are defined as −∞ and ∞ , respectively. This definition ensures that $t_{\mathrm{low}}\geq 0$ and $t_{\mathrm{up}}$ is less or equal the maximum observed event time. The hazard in the interval is estimated as the ratio of the number of events and the sum of all observed times, that is, ${\hat{\lambda }}_{i}(q_{i}(\gamma ))=(\left(N_{i}(t_{\mathrm{up}})-N_{i}(t_{\mathrm{low}})\right)/\left(\int_{t_{\mathrm{low}}}^{t_{\mathrm{up}}}Y_{i}(s)ds\right)$ .

With an increasing number of events, the interval becomes narrower while the absolute number of events within the interval gets larger. Under the assumption of continuous hazard function and by the consistency of ${\hat{q}}_{i}(\gamma )$ , the resulting estimate is consistent. In the actual calculations, we used K=2 . Alternatively, the local hazard may be estimated by kernel-density estimation.^28^

When the ratio of quantiles is of interest, the difference at the log scale may be defined as a parameter of interest such that ${\hat{\theta }}_{j}=\log\;{\hat{q}}_{1}(\gamma )-\log\;{\hat{q}}_{0}(\gamma )$ . By application of the delta method to the original approximation, we obtain the required presentation as

$$\begin{array}{r} \log\;{\hat{q}}_{i}(\gamma )-\log\;q_{i}(\gamma )\approx \frac{-1}{{\hat{q}}_{i}(\gamma ){\hat{\lambda }}_{i}(q_{i}(\gamma ))}\int_{0}^{t}\frac{1}{Y_{i}(s)}dM(s) \end{array}$$

#### 2.2.3. Average hazard ratio

A general class of average hazard ratios can be defined as $\int_{0}^{L}W(s)d{\hat{\Lambda }}_{1}(s)/\int_{0}^{L}W(s)d{\hat{\Lambda }}_{0}(s)$ , where L is a predefined time point and W(s),s≥0 is a non-negative monotonically decreasing weight function with values in [0,1] .^18^ We consider the average hazard ratio with weight function $W(s)={\hat{S}}_{0}(s){\hat{S}}_{1}(s)$ and its corresponding estimate $\hat{W}(s)={\hat{S}}_{0}^{-}(s){\hat{S}}_{1}^{-}(s)$ . Here the left continuous estimator of the survival function is used to obtain a predictable function, which is required in the eventual application of the martingale central limit theorem. Note that both $\hat{S}$ and ${\hat{S}}^{-}$ are uniformly consistent estimates of S , and with sufficient sample size their numeric difference is negligible.

The specific weight function was chosen, because the average hazard ratio based on the this weight function is identical to $P(T_{1}\wedge L>T_{0}\wedge L)/P(T_{1}\wedge L<T_{0}\wedge L)$ and can be interpreted as a non-parametric effect measure similar to a Mann–Whitney statistic. Unlike the Cox model hazard ratio estimate, the limiting value of this average hazard ratio estimate does not depend on the censoring distribution, because it is entirely defined through the survival functions, which can be estimated consistently regardless of the pattern of random censoring times under the assumption that censoring times and survival times are independent. (Also see simulation scenarios 2 and 3 in Section 4.).

To embed the average hazard ratio in the proposed framework we utilize the log-transformed estimate ${\hat{\theta }}_{j}=\log\;(\int_{0}^{L}\hat{W}(s)d{\hat{\Lambda }}_{1}(s))-\log\;(\int_{0}^{L}\hat{W}(s)d{\hat{\Lambda }}_{0}(s))$ .

The contribution of group i to the estimate of the log-average hazard ratio immediately fits into the proposed framework as $\int_{0}^{L}\hat{W}(s)d{\hat{\Lambda }}_{i}(s)-\int_{0}^{L}\hat{W}(s)d\Lambda_{i}(s)=\int_{0}^{L}\hat{W}(s)(1/Y_{i}(s))dM_{i}(s)$ .

By application of the delta method, the representation according to (1) for the log-transformed term is

$$\begin{array}{r} \log\;\left(\int_{0}^{L}\hat{W}(s)d{\hat{\Lambda }}_{i}(s)\right)-\log\;\left(\int_{0}^{L}\hat{W}(s)d\Lambda_{i}(s)\right)\\ \approx \frac{1}{\int_{0}^{L}\hat{W}(s)d{\hat{\Lambda }}_{i}(s)}\int_{0}^{L}\hat{W}(s)\frac{1}{Y_{i}(s)}dM_{i}(s) \end{array}$$

Note that the statistic $\sqrt{n_{i}}\int_{0}^{L}\hat{W}(s)(1/Y_{i}(s))dM_{i}(s)$ is asymptotically equivalent to $\sqrt{n_{i}}\int_{0}^{L}W(s)(1/Y_{i}(s))dM_{i}(s)$ . This follows from the following arguments: By the martingale central limit theorem, the difference between both expressions, $\sqrt{n_{i}}\int_{0}^{L}\hat{W}(s)(1/Y_{i}(s))dM_{i}(s)-\sqrt{n_{i}}\int_{0}^{L}W(s)(1/Y_{i}(s))dM_{i}(s)=\sqrt{n_{i}}\int_{0}^{L}(\hat{W}(s)-W(s))(1/Y_{i}(s))dM_{i}(s)$ , converges in distribution to a normal distribution with mean zero and variance $\int_{0}^{L}(\hat{W}(s)-W(s){)}^{2}(n/Y(s))d\Lambda (s)$ . Because ${\hat{S}}_{i}^{-}$ and, consequently, $\hat{W}(s)$ are uniformly strongly consistent estimators, the variance of $\sqrt{n_{i}}\int_{0}^{L}(\hat{W}(s)-W(s))(1/Y_{i}(s))dM_{i}(s)$ can be bounded by $\int_{0}^{L}(\hat{W}(s)-W(s){)}^{2}(n/Y(s))d\Lambda (s)\leq \max_{0\leq s\leq L}((\hat{W}(s)-W(s){)}^{2})\int_{0}^{L}(n_{i}/Y(s))d\Lambda (s)$ . Since $\max_{0\leq s\leq L}((\hat{W}(s)-W(s){)}^{2})$ converges to 0 a.s. and $\int_{0}^{L}(n_{i}/Y(s))d\Lambda (s)$ converges to a constant (the asymptotic variance of ${\hat{\Lambda }}_{i}(L)$ ), the variance of $\sqrt{n_{i}}\int_{0}^{L}(\hat{W}(s)-W(s))(1/Y_{i}(s))dM_{i}(s)$ converges to 0. Hence, the asymptotic arguments established in Section 2.1 are not affected by using estimated weights $\hat{W}$ instead of the true (unknown) weights W and $\int_{0}^{L}\hat{W}(s)(1/Y_{0}(s))dM_{0}(s)$ and $\int_{0}^{L}\hat{W}(s)(1/Y_{1}(s))dM_{1}(s)$ are asymptotically uncorrelated.

#### 2.2.4. Restricted mean survival time (RMST)

The RMST in group i up to a pre-specified time-point L is $\mu_{i}=\int_{0}^{L}S_{i}(t)dt$ . Let $D_{i}$ be the number of unique event times $t_{i,1}<\ldots <t_{i,D_{i}-1}\leq L$ in group i that are less or equal L . Further define $t_{i,0}=0$ and $t_{i,D_{i}+1}=L$ and $\Delta t_{i,j}=t_{i,j+1}-t_{i,j}$ . The according estimate for the RMST is ${\hat{\mu }}_{i}=\sum_{j=0}^{D_{i}}{\hat{S}}_{i}(t_{j})\Delta t_{i,j}$ and the estimated RMST difference between the two groups is ${\hat{\theta }}_{j}={\hat{\mu }}_{1}-{\hat{\mu }}_{0}$ . ${\hat{\mu }}_{i}$ may be represented in terms of equation (1).^29,30^ First note that

$$\begin{array}{rl} {\hat{\mu }}_{i}-\mu_{i} & =\sum_{j=0}^{D_{i}}{\hat{S}}_{i}(t_{j})\Delta t_{i,j}-\sum_{j=0}^{D_{i}}S_{i}(t_{j})\Delta t_{i,j}+\sum_{j=0}^{D_{i}}S_{i}(t_{j})\Delta t_{i,j}-\int_{0}^{L}S_{i}(t)dt\\ & =\sum_{j=0}^{D_{i}}\Delta t_{i,j}({\hat{S}}_{i}(t_{j})-S_{i}(t_{j}))+o_{p}(1/\sqrt{n_{i}}) \end{array}$$

where we use that the error of the integral approximation is of order 1/n . By replacing ${\hat{S}}_{i}(t_{j})-S_{i}(t_{j})$ by equation (5) and by changing the order of integration and summation the required form is obtained as

$$\begin{array}{r} {\hat{\mu }}_{i}-\mu_{i}\approx -\int_{0}^{L}\left(\sum_{j\in \{1,\ldots ,D_{i}:t_{j}\geq s\}}\Delta t_{j}S_{i}(t_{j})\right)(1/Y_{i}(s))dM_{i}(s) \end{array}$$

Analysing RMST has been proposed as an alternative to hazard ratio-based inference in different medical fields such as oncology^31,32^ and cardiology.^33^ As an extension to between-group comparisons considered here, RMST is amenable to regression analysis, see Hasegawa et al.^29^ for a recent review. Also, group sequential methods have been developed.^34^

#### 2.2.5. Cox model score test (logrank test)

The logrank test for the null hypothesis $H_{0}:\lambda_{0}(s)=\lambda_{1}(s),\forall 0\leq 0\leq L$ may be of interest also under non-proportional hazard settings. The usual logrank test is asymptotically equivalent to the Cox model score test for the null hypothesis β=0 , where β is the Cox model hazard ratio. The tests are equivalent to the variance estimate for the score test statistic. The score test may be directly included in the proposed framework to adjust for multiple testing as shown below.

For subject $j=1,\ldots ,n_{i}$ in group i∈{0,1} , let $y_{ij}(t)\in \{0,1\}$ indicate whether the subject is at risk at time t , and let $N_{ij}(t)\in \{0,1\}$ be the number of events of the subject in the time interval [0,t] . Let $M_{ij}(t)=N_{ij}(t)-y_{ij}(t)\Lambda_{i}(t)$ .

In a Cox model comparing two treatment groups up to a time point L , the score function (i.e. the derivative of the log partial likelihood) is

(6) $$U(\beta )=\sum_{i=0}^{1}\sum_{j=1}^{n_{i}}\int_{0}^{L}\left(i-\frac{Y_{1}(s)\exp\;(\beta )}{Y_{0}(s)+Y_{1}(s)\exp\;(\beta )}\right)dN_{ij}(s)$$

It can be shown^35^ that under the proportional hazards assumption (i.e. $\lambda_{1}(t)=\lambda_{0}(t)\exp\;(\beta ),\forall t\geq 0$ ), and for $\beta =\beta_{0}$ being the true parameter value, $dN_{ij}$ may be replaced by $dM_{ij}$ , such that

(7) $$U(\beta_{0})=\sum_{i=0}^{1}\sum_{j=1}^{n_{i}}\int_{0}^{L}\left(i-\frac{Y_{1}(s)\exp\;(\beta_{0})}{Y_{0}(s)+Y_{1}(s)\exp\;(\beta_{0})}\right)dM_{ij}(s)$$

which can be rewritten as

(8) $$U(\beta_{0})=\int_{0}^{L}\frac{Y_{0}(s)Y_{1}(s)}{Y_{0}(s)+Y_{1}(s)\exp\;(\beta_{0})}\frac{1}{Y_{1}(s)}dM_{1}(s)-\int_{0}^{L}\frac{Y_{0}(s)Y_{1}(s)\exp\;(\beta_{0})}{Y_{0}(s)+Y_{1}(s)\exp\;(\beta_{0})}\frac{1}{Y_{0}(s)}dM_{0}(s)$$

Note that under the null hypothesis $H_{0}:\lambda_{0}(s)=\lambda_{1}(s),\forall s\leq 0\leq L$ , the proportional hazard assumption holds. Further note that rejection of β=0 entails rejection of $\lambda_{0}(s)=\lambda_{1}(s)$ at least for some s . The reverse is not necessarily true under crossing hazards, that is, the hazard functions $\lambda_{0}(s)$ and $\lambda_{1}(s)$ may be different but crossing in such a way that β=0 , which results in low power of the logrank test under crossing hazards. The test statistic for the score test of the null-hypothesis $\beta =\beta_{0}$ is $U(\beta_{0})$ as defined in 8. Hence, the Cox model score test for the null hypothesis β=0 can be included in the framework of Section 2.1 by defining a parameter estimate ${\hat{\theta }}_{j}=U(0)$ and by setting $H_{k,i}(s)=\left(Y_{0}(s)Y_{1}(s)\right)/\left(Y_{0}(s)+Y_{1}(s)\right)$ and $a_{k,i}=1$ in equation (1).

Note that Assumption 3 about uncorrelated contributions from both groups is satisfied for the score test despite both terms in equation (8) containing the at-risk process of both groups, because we assume that the probability for equal event times is 0 (and ties only occur due to rounding of observed event times). Under this assumption, Theorems 2.5 (Section 2) and 2.4 (Section 4) of Fleming and Harrington^8^ show that the covariance of statistics of the type assumed in equation (1) is 0.

#### 2.2.6. Cox model hazard ratio

The Cox model hazard ratio can be included in the proposed framework of simultaneous inference under the assumption of proportional hazards. First note that the estimate of the log hazard ratio, $\hat{\beta }$ , is the solution of $U(\hat{\beta })=0$ . Next, by standard asymptotic results and with $\beta_{0}$ the true log hazard ratio $\hat{\beta }-\beta_{0}\approx -(dU/d\beta {)}^{-1}(\beta_{0})U(\beta_{0})$ .^36^ Here, $U(\beta_{0})$ can be decomposed in contributions from the two groups according to (8). The Hessian matrix H=dU/dβ can be estimated by

$$\begin{array}{r} \hat{H}=-\sum_{i=0}^{1}\int_{0}^{L}\frac{Y_{0}(s)Y_{1}(s)\exp\;(\hat{\beta })}{Y_{0}(s)+Y_{1}(s)\exp\;(\hat{\beta })}dN_{i}(s) \end{array}$$

Therefore, the Cox model hazard ratio can be included in the framework of Section 2.1, by setting $\theta_{j}=\hat{\beta }$ , $H_{k,i}(s)=\left(Y_{0}(s)Y_{1}(s)\exp\;(i\hat{\beta })\right)/\left(Y_{0}(s)+Y_{1}(s)\exp\;(\hat{\beta })\right)$ and $a_{k,i}=(-\hat{H}{)}^{-1}$ . The theory of Section 2.1 applies to the hazard ratio under the assumption of proportional hazards. The operating characteristics of simultaneous inference including the hazard ratio under non-proportional hazards are explored in the Supplemental material.

### 2.3. Resampling-based covariance matrix estimate

As an alternative method, the covariance matrix for a vector of parameter estimates $\hat{\theta }=({\hat{\theta }}_{1},\ldots ,{\hat{\theta }}_{m})$ may be estimated by a resampling approach similar to perturbation methods described by Zhao et al.^30,37^ and Parzen et al.,^38^ which can be regarded as parametric bootstrap.

Based on the martingale central limit theorem and equation (2), the process ${\hat{\Lambda }}_{i}(t)-\Lambda_{i}(t)=\int_{0}^{t}(1/Y_{i}(s))dM_{i}(s),t\geq 0$ can be approximated by a Gaussian process with independent increments and covariance function $\mathrm{cov}({\hat{\Lambda }}_{i}(t)-\Lambda_{i}(t),{\hat{\Lambda }}_{i}(t^{\prime })-\Lambda_{i}(t^{\prime }))=\mathrm{var}({\hat{\Lambda }}_{i}(t)-\Lambda_{i}(t))=\int_{0}^{t}(dN_{i}(s)/Y_{i}^{2}(s))$ . Accordingly, the distribution of this process can be approximated by the distribution of the process $P(t)=\sum_{j=1}^{n_{i}}\int_{0}^{t}Z_{j}\sqrt{\left(dN_{ij}(s)/Y_{i}^{2}(s)\right)},t\geq 0$ , where $Z_{j},j=1,\ldots ,n_{i}$ are independent standard normal random variables.

A perturbation sample of $\hat{\Lambda }-\Lambda$ is defined as $P^{*}(t)=\sum_{j=1}^{n_{i}}\int_{0}^{t}z_{j}\sqrt{(dN_{ij}(s)/Y_{i}^{2}(s))},t\geq 0$ , where $z_{j},j=1,\ldots ,n_{i}$ are realizations of independent standard normal random variables. And a perturbation of $\hat{\Lambda }$ is defined as ${\hat{\Lambda }}^{*}=\hat{\Lambda }+P^{*}$ . Similar to equation (2), in the case of ties, the expression $dN_{ij}(s)/Y_{i}^{2}(s)$ may be replaced by (3) if $dN_{i}(s)\geq 1$ .

A vector of parameter estimates can be regarded as function $\hat{\theta }({\hat{\Lambda }}_{0},{\hat{\Lambda }}_{1})$ of the estimated cumulative hazard functions. In the proposed perturbation approach, the distribution of $\hat{\theta }({\hat{\Lambda }}_{0},{\hat{\Lambda }}_{1})$ given the true cumulative hazard functions $\Lambda_{0}$ and $\Lambda_{1}$ is approximated by the distribution of $\hat{\theta }({\hat{\Lambda }}_{0}^{*},{\hat{\Lambda }}_{1}^{*})$ given ${\hat{\Lambda }}_{0}$ and ${\hat{\Lambda }}_{1}$ .

To estimate the covariance matrix of $\hat{\theta }({\hat{\Lambda }}_{0},{\hat{\Lambda }}_{1})$ , a large number of K perturbation pairs $({\hat{\Lambda }}_{0}^{*},{\hat{\Lambda }}_{1}^{*}{)}_{l},l=1,\ldots ,K,$ is generated and for each pair the estimate ${\hat{\theta }}_{l}^{*}=\hat{\theta }(({\hat{\Lambda }}_{0}^{*},{\hat{\Lambda }}_{1}^{*}{)}_{l}),$ is calculated. Now consider the matrix $\left({\hat{\theta }}_{1}^{*},\ldots ,{\hat{\theta }}_{K}^{*}\right)$ and let ${\hat{\Theta }}^{*}$ be the corresponding row-mean centred matrix. Then the covariance matrix of $\hat{\theta }$ can be estimated by the empirical covariance matrix as

(9) $${\hat{\text{cov}}}_{pert}(\hat{\theta })={\hat{\Theta }}^{*}{\hat{\Theta }}^{*T}/(K-1)$$

## 3. Simultaneous inference

### 3.1. Maximum-type hypothesis tests for multivariate normal statistics

We aim to test the hypothesis with respect to parameters $\theta =(\theta_{1},\ldots ,\theta_{m})$ , as defined in the previous sections, with strong control of the family-wise type I error rate. We consider one-sided elementary null hypotheses $H_{k}:\theta_{k}\leq \theta_{k}^{\left(0\right)}$ , k=1,…,m and the global intersection null hypothesis of no difference in any of the parameters $H_{0}=\cap_{k=1}^{m}H_{k}$ . In superiority trials, depending on whether the parameter of interest is ratios or differences between treatment and control, $\theta_{k}^{\left(0\right)}$ is set to 1 or 0, respectively. The considered hypothesis tests can also be defined as two-sided hypotheses, but for the purpose of showing the superiority of treatment over a control we regard one-sided tests to be more relevant.

Multiple hypothesis tests can be constructed based on the multivariate normal approximation of the estimates $\hat{\theta }=({\hat{\theta }}_{1},\ldots ,{\hat{\theta }}_{m})$ , see, for example, Hothorn et al.^39^ Correlations of estimates can be high, hence the multiplicity correction based on the approximate joint distribution can be moderate compared to methods that control the family-wise type I error rate without taking into account the correlation structure, as the Bonferroni correction. We consider maximum-type tests. Other test statistics, such as sum or chi-squared type statistics may also be used within the multivariate normal framework. Consider a vector of m parameter estimates $\hat{\theta }\overset{\mathrm{appr}.}{∼}N_{m}(\theta ,\hat{\Sigma })$ . Let $\hat{V}$ be the diagonal matrix with diagonal entries of $\hat{\Sigma }$ . Define a vector of elementary test statistics $T={\hat{V}}^{-1/2}(\hat{\theta }-\theta_{0})$ . Under the global null hypothesis $H_{0}:\theta =\theta_{0},T\overset{\mathrm{appr}.}{∼}N(0,{\hat{V}}^{-1/2}\hat{\Sigma }{\hat{V}}^{-1/2})$ . A one-sided p -value for the test of $H_{0}$ is given by P{max(Z)≥max(T)} , where $Z∼N_{m}(0,{\hat{V}}^{-1/2}\hat{\Sigma }{\hat{V}}^{-1/2})$ .

A single-step maximum-type test for the elementary null hypothesis can then be defined via multiplicity-adjusted p -values for the hypotheses $H_{k},k\in \{1,\ldots ,m\}$ and is given by $P\{\max(Z)\geq T_{k}\}$ .

The single-step test can be improved by applying the closed testing procedure,^40^ where each intersection null hypothesis $\cap_{k\in I}H_{k},I\subset 1,\ldots ,m$ is tested by a maximum-type test for intersection hypotheses. The elementary hypothesis $H_{k}$ is then rejected by the closed test, if all intersection hypotheses that include $H_{k}$ (i.e. all $\cap_{k\in I}H_{k}$ with I⊂1,…,m and k∈I ) are rejected with the corresponding maximum-type test. A multiplicity-adjusted p -value is given by the maximum of the elementary p -values of these intersection hypotheses.

### 3.2. Simultaneous confidence intervals

Lower confidence bounds with simultaneous coverage probability 1−α that correspond to the inversion of a one-sided single-step multiple-testing procedure are given by ${\hat{\theta }}_{j}-{\hat{V}}_{j}^{1/2}q_{\alpha }$ , where $q_{\alpha }$ is defined as $q_{\alpha }:P(\max(Z)\geq q_{\alpha })=\alpha$ . The boundaries of two-sided confidence intervals with simultaneous coverage probability 1−α that correspond to the inversion of a two-sided single-step test are defined as ${\hat{\theta }}_{j}\pm {\hat{V}}_{j}^{1/2}q_{\alpha }^{\prime }$ , where $q_{\alpha }^{\prime }$ is defined as $q_{\alpha }^{\prime }:P(\max(|Z|)\geq q^{\prime })=\alpha$ .

In an actual analysis, the closed testing procedure may be preferred over the single-step procedure as it is more powerful. It is possible to construct confidence regions that correspond to the inversion of the closed testing procedure. However, such regions would typically be of complex shape and not easily interpreted, and the projections of such a region onto univariate confidence intervals would typically not retain the advantage of the closed test over the single-step test.^41^

## 4. Simulation study

In six different simulation scenarios and for different parameter sets, we studied the operating characteristics of the proposed multivariate inference methods and compared them with the Bonferroni–Holm multiplicity adjustment and a single logrank test.

We considered seven parameter sets. In parameter set 1, we included the average hazard ratio and the restricted mean survival time as an example of joint inference using two alternative summary measures of a possible treatment benefit.

In parameter set 2, we included the between-group difference in survival probabilities after 1, 2 and 3 years as well as 25% quantiles and the restricted mean survival time. This parameter set was intended to illustrate an analysis where one summary measure (RMST) is combined with several statistics that allow a point-wise description of the survival differences. Parameter set 3 was defined similarly, albeit replacing survival probabilities and quantiles by their log-transformations. This set was included to study a possible effect of log transformation on the finite sample properties of the distributional approximation. In a similar spirit, parameter set 4 included cloglog-transformed survival probabilities combined with the average hazard ratio. The comparison of untransformed, log-transformed and cloglog-transformed survival differences is of particular interest as the asymptotic normal approximation may work differently well with small sample sizes depending on the chosen transformation.

Parameter sets 5 to 7 were designed to study simultaneous inference for the logrank test combined with estimators for several parameters that quantify differences between the survival functions. Parameter sets 5 and 6 included the logrank test and differences in untransformed or cloglog-transformed survival probabilities, respectively. In parameter set 7, we combined the logrank test with estimates for the average hazard ratio and restricted mean survival time.

In all scenarios, a cut-off value of 3 years was set for the restricted mean survival time, for (average) hazard ratios and for the calculation of the logrank test. The rationale for this approach was to have an interpretable comparison between the logrank test on the one side and RMST and average hazard ratio on the other side. By applying the same cut-off value to all methods, they use the same data and the results are comparable. If the logrank test was used without cut-off it would be difficult to say if power differences are due to more data being available to the logrank test or due to inherent power differences.

Table 2 shows a summary of the considered parameter sets and the true values of the respective parameters in the simulation scenarios. Further simulation scenarios, including also the Cox model hazard ratio (despite its limitation) are considered in the Supplemental material.

**Table 2.** Parameter sets in the simulation study. All parameters considered in the simulation are listed and their true values are shown for scenarios 1 to 6 of Section 4.1. For log-scaled parameters of Section 2.2, back-transformed values, that is, ratios, are shown in the table. The expected value of the Cox model HR estimate is shown for comparison. For the score test, the expected contribution of an individual to the summed score statistic is shown. Cross-marks (x) indicate which parameter is included in which set.

			Scenario						Parameter set						
Parameter	Interpretation	Time	1	2	3	4	5	6	1	2	3	4	5	6	7
S	Survival diff.	1	0.00	−0.06	−0.06	0.12	0.12	0.17		x			x		
S		2	0.16	0.16	0.16	0.15	0.10	0.15		x			x		
S		3	0.20	0.29	0.29	0.15	0.09	0.10		x			x		
logS	Survival ratio	1	1.00	0.92	0.92	1.19	1.34	1.35			x				
logS		2	1.41	1.43	1.43	1.42	1.63	1.70			x				
logS		3	2.28	3.29	3.29	1.69	1.74	2.05			x				
Q	25% qu. diff.		0.10	-0.26	-0.26	0.31	0.09	0.29		x					
logQ	25% qu. ratio		1.09	0.74	0.74	1.54	1.29	1.71			x				
RMST	RMST diff.	3	0.26	0.20	0.20	0.36	0.28	0.40	x	x	x				x
cloglogS	Cumulative HR	1	1.00	1.28	1.28	0.65	0.73	0.60				x		x	
cloglogS		2	0.63	0.64	0.64	0.65	0.73	0.65				x		x	
cloglogS		3	0.56	0.43	0.43	0.65	0.74	0.69				x		x	
avgHR	Average HR	3	0.67	0.75	0.75	0.65	0.74	0.62	x			x			x
HR	Cox model HR	3	0.63	0.62	0.69	0.65	0.73	0.64							
score	Score test	3	-0.07	-0.07	-0.05	-0.06	-0.06	-0.08					x	x	x

HR: hazard ratio; avgHR: average HR: RMST: restricted mean survival time; cloglogS: complementary log–log scenario.

To assess the coverage probabilities of confidence intervals, simulations were performed for parameter sets 1 to 4 (see Table 2) with sample sizes 50, 100, 200 or 1000 per group. Adjusted and unadjusted two-sided confidence intervals with a nominal (simultaneous) coverage probability of 95% were calculated. The asymptotic covariance matrix estimate and the perturbation-based estimate, both with adjustment for ties, were used.

To assess type I error rate and power of hypothesis tests, simulations were performed for parameter sets 5 to 7 (see Table 2) with a sample size of 50 and 200 per group, under the alternative and under the null hypothesis of identical survival curves. In the latter case, data for both groups was sampled from the distribution of the control group. For each parameter set $\left(\theta_{1},\ldots ,\theta_{m}\right)$ , the elementary null hypotheses $H_{1}:\theta_{1}=0,\ldots ,H_{m}:\theta_{m}=0$ versus one-sided alternatives were considered, as well as the global null hypothesis $\cap_{i=1}^{m}H_{i}$ . The null hypotheses were tested with the closed testing procedure described in Section 3 and, for comparison, with unadjusted and with Bonferroni–Holm adjusted tests where the elementary p -values were computed based on univariate normal approximations. The nominal (family-wise) one-sided significance level was set to 0.025. To calculate the critical values, the asymptotic covariance matrix estimate with adjustment for ties was used (see Section 2.1). Simulation results using the resampling-based covariance estimate (see Section 2.3) are given in the Supplemental material.

All simulations were repeated 50,000 times. The resulting simulation standard error for type I error rate, power or confidence interval coverage can be assessed from the normal approximation to the binomial distribution. It is at most 0.22 percentage points. Assuming a true type I error rate of 2.5% one-sided and a true confidence interval coverage of 95% two-sided, respective simulation standard errors are 0.07 or 0.10 percentage points only. However, with a sample size of 50 per group under scenario 1, in 24 out of 50,000 runs, the variance of the 1-year survival probability was not estimable due to zero events within 1 year in one group. These runs were excluded from the calculations.

### 4.1. Simulation scenarios

In all scenarios, we compare two groups with an equal number of patients per group in (50,100,200,1000) . Recruitment was assumed to be uniform over 1 or 1.5 years (depending on the scenario) and the maximum follow-up time was 3.5 years in all scenarios. Furthermore, we applied random censoring according to an exponential distribution with rate −log(0.9) such that, given no other events occur, on average within 1 year 10% and within 2 years 19% of subjects are censored. Simulated survival times were rounded to full days to reflect the degree of precision and the occurrence of ties as observed in actual trials.

**Figure 1.**
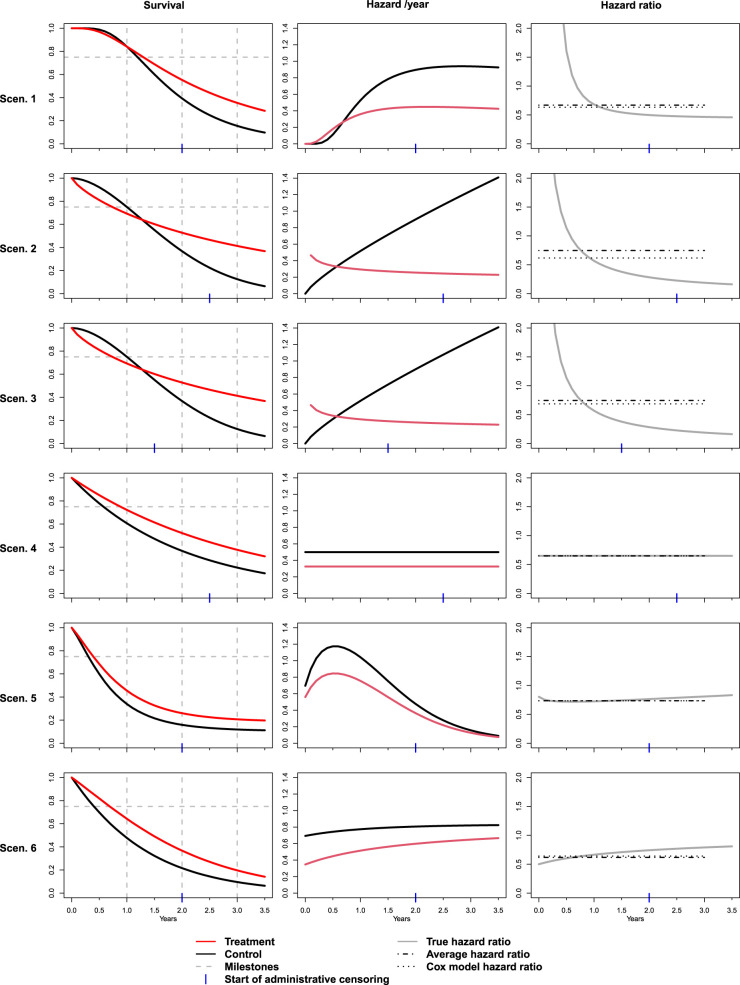
Simulation scenarios. Left column: True survival functions for treatment and control group. Vertical dashed lines indicate the 1-, 2- and 3-year time point. The horizontal line indicates the 25% quantile. Middle column: True hazard rates (per year) as a function of time for treatment and control group. Right column: Hazard ratio between treatment and control as a function of time. Dotted and dash-dotted lines indicate the large sample limit of the Cox model hazard ratio estimate and the true average hazard ratio as defined in Section 2.2, both calculated with a cut-off at 3 years.

The scenarios are described in detail below, and the resulting survival functions, hazard functions and hazard ratios as a function of time are shown in Figure 1.

**Scenario 1, delayed onset of treatment effect:** In Scenario 1, we sampled data for the treatment group from a lognormal $\left(0.8,0.8^{2}\right)$ distribution and data for the control group from a lognormal $\left(\log\;(\exp\;(0.8)-0.5),(\log\;(\exp\;(0.8)-0.5){)}^{2}\right)$ distribution. The distributions were chosen to resemble a setting with a delayed onset of the treatment effect. During the initial phase, the control group has slightly better survival, which may occur if the treatment effect is observed only after a certain duration of treatment, but the potential risk of side effects increases immediately after treatment starts. The recruitment phase in the simulation was 1.5 years.

**Scenario 2, crossing survival curves, fast recruitment:** In Scenario 2, we used Weibull(2,1.8) and Weibull(3.5,0.8) distributions (where parameters refer to scale and shape) for the treatment and the control group, respectively. The resulting distributions, as illustrated in Figure 1, show a pronounced crossing of the survival functions and the hazard functions. The assumed duration of the recruitment phase was 1 year.

**Scenario 3, crossing survival curves, slow recruitment:** Scenario 3 was identical to scenario 2, except that the duration of the recruitment phase was 2 years, which resulted in a modified censoring pattern.

**Scenario 4, proportional hazards:** In Scenario 4, data are sampled from Exp(0.5) and Exp( 0.5⋅0.65 ) distributions, meeting the proportional hazards assumption with a hazard ratio of 0.65, with a recruitment phase of 1 year.

**Scenario 5, cure fraction:** In scenario 5, we assumed that 30% of patients belong to a subpopulation of strong treatment responders in whom the treatment leads to a complete cure and we further assumed that in the control group, patients are switched to active treatment after disease progression with 70% probability. Transitions to progression or death were assumed to be governed by independent processes with constant rates. Hazard rates for death were 0.69 per year before progression and 2.77 per year after progression and the progression rate was 1.39 per year. (This corresponds to median event times for the three processes of 12, 6 and 3 months, respectively.) The recruitment phase was 1.5 years. Data simulation for scenarios 5 and 6 was performed using The R library nph.^5^

**Scenario 6, rescue medication:** In the final scenario, we assume that a rescue medication is applied to all patients in both groups after disease progression. The assumed hazard rates per year for death were 0.35 under treatment, 0.69 under control and 0.83 under rescue medication (corresponding to median times of 24, 12 and 10 months). Progression rates were 0.52 under treatment and 0.92 under control (corresponding to median times of 16 and 9 months). The recruitment phase was 1.5 years.

### 4.2. Simulation results

**Figure 2.**
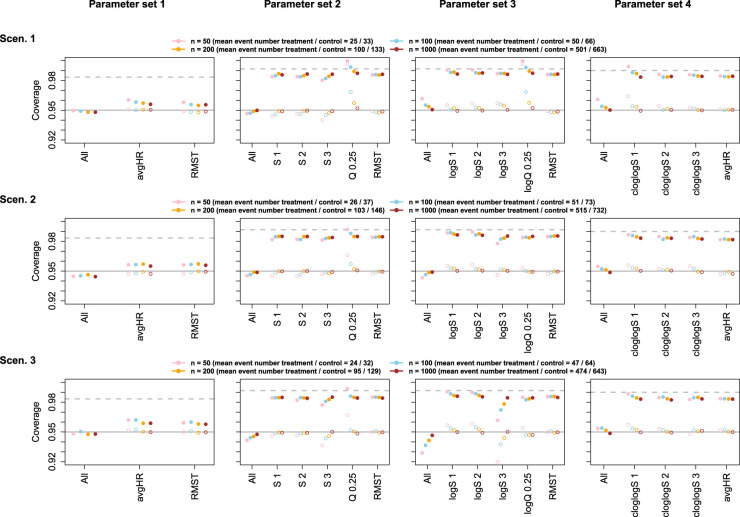
Empirical coverage of confidence intervals for scenarios 1–3, based on the multivariate normal distribution with asymptotic covariance matrix estimate. Filled circles show the simultaneous coverage (All) and univariate coverage probability of multiplicity-adjusted intervals for single parameters (abbreviations as in Table 2). Open circles represent the coverage of unadjusted univariate confidence intervals. Error bars represent 95% Wald confidence intervals for the respective coverage probabilities. For comparison, the horizontal solid line indicates the nominal coverage of 95%, and the horizontal dashed line indicates the univariate confidence level that would result from a Bonferroni adjustment for the respective number of parameters. S 1: scenario 1; S 2: scenario 2; S 3: scenario 3; avgHR: average hazard ratio; RMST: restricted mean survival time; logS 1; log scenario 1; logS 2; log scenario 2; logS 3; log scenario 3; cloglogS 1: complementary log–log scenario 1; cloglogS 2: complementary log–log scenario 2; cloglogS 3: complementary log–log scenario 3.

**Figure 3.**
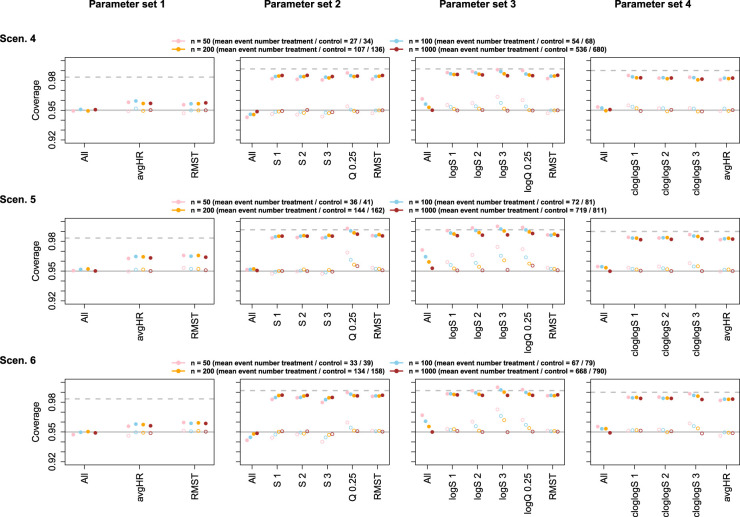
Empirical coverage of confidence intervals for Scenarios 4–6, based on the multivariate normal distribution with asymptotic covariance matrix estimate. Further details as in Figure 2. S 1: scenario 1; S 2: scenario 2; S 3: scenario 3; avgHR: average hazard ratio; RMST: restricted mean survival time; logS 1; log scenario 1; logS 2; log scenario 2; logS 3; log scenario 3; cloglogS 1: complementary log–log scenario 1; cloglogS 2: complementary log–log scenario 2; cloglogS 3: complementary log–log scenario 3.

The empirical coverage probabilities of simultaneous confidence intervals as well as of unadjusted, univariate confidence intervals were in general close to the nominal 95% with sizes of 200 or 1000 per group (and approximately half as many events), for both the asymptotic and the perturbation covariance matrix estimate. With sample sizes of 50 or 100 per group, deviations from the nominal coverage were mostly below one percentage point. Stronger deviations due to small sample sizes were observed when including log-transformed survival probabilities, as opposed to untransformed or cloglog-transformed survival probabilities, with the asymptotic covariance matrix estimate. In contrast, intervals for the difference in cloglog-transformed survival were overly conservative with the perturbation approach applied to small sample sizes, in particular for early time points. In general, the perturbation method was less robust with respect to small sample sizes than methods using the asymptotic covariance matrix estimate, however, deviations led to conservative tests in almost all cases. For both methods, deviations from the nominal coverage were not specific to the simultaneous inference methods but were also observed for the univariate confidence intervals. Results for the asymptotic covariance estimate are shown in Figures 2 and 3, results for the perturbation-based covariance estimate are shown in Figures S1 and S2 in the Supplemental material. The univariate coverage of multiplicity-adjusted intervals was >95%, but the intervals were less conservative than simple Bonferroni-adjusted intervals. Similar results with close to nominal coverage under sufficient sample sizes were observed for parameter sets including the Cox model hazard ratio, see Figures S3 and S4 in the Supplemental material.

With a sample size of 200 per group, the type I error rate of one-sided unadjusted hypothesis tests for the studied parameters was well controlled at the 2.5% level with the exception of tests for untransformed survival probabilities. However, it is well known that the normal approximation for untransformed survival probabilities may not be entirely appropriate with small sample sizes. In contrast, tests for cloglog-transformed survival did control the type I error rate. See column ‘T1E unadj’ in Tables 3 and 4.

**Table 3.** T1E and Pow for unadj tests, multiplicity adjustment through the multivariate normal-based closed test (adj) and Holm tests, observed in 50,000 simulation runs for scenarios 1–3. Rows labelled ‘Any’ refer to the probability of rejecting the null hypothesis for at least one included parameter (i.e. family-wise T1E or Pow). Further rows show the rejection probability for each specific parameter included in the parameter set. The sample size in the simulation was 200 subjects per group. Values are presented as per cent.

Scenario	Set	Parameter	T1E unadj	T1E adj	T1E Holm	Pow unadj	Pow adj	Pow Holm
1	5	Any	7.56	2.73	2.21	97.2	92.5	91.1
		S 1	2.58	0.92	0.75	2.6	2.5	2.5
		S 2	2.54	0.92	0.76	84.7	76.6	75.4
		S 3	2.59	0.95	0.79	92.3	86.2	85.2
		Score test	2.44	0.80	0.64	92.9	86.3	84.4
	6	Any	7.13	2.38	1.87	97.1	92.2	90.5
		cloglogS 1	2.42	0.78	0.60	2.4	2.3	2.3
		cloglogS 2	2.48	0.86	0.71	84.4	75.9	74.6
		cloglogS 3	2.31	0.74	0.60	92.1	85.5	84.4
		Score test	2.44	0.79	0.62	92.9	86.2	84.2
	7	Any	3.22	2.46	1.01	92.9	91.1	84.7
		avgHR	2.54	2.10	0.96	84.8	83.8	78.1
		RMST	2.54	2.10	0.95	83	82.4	77.8
		Score test	2.44	2.00	0.96	92.9	91.0	84.7
2	5	Any	7.41	2.65	2.13	100.0	99.9	99.9
		S 1	2.6	0.89	0.71	0.1	0.1	0.1
		S 2	2.58	0.91	0.75	86.0	79.6	78.2
		S 3	2.51	0.87	0.73	100.0	99.9	99.9
		Score test	2.46	0.90	0.71	95.3	91.3	89.6
	6	Any	7.09	2.39	1.90	100.0	99.9	99.9
		cloglogS 1	2.47	0.79	0.61	0.1	0.1	0.1
		cloglogS 2	2.5	0.86	0.70	85.7	79.1	77.7
		cloglogS 3	2.31	0.71	0.60	100.0	99.9	99.9
		Score test	2.46	0.89	0.71	95.3	91.3	89.6
	7	Any	3.38	2.52	1.14	95.3	93.8	89.0
		avgHR	2.63	2.11	1.04	60.6	58.1	49.6
		RMST	2.62	2.11	1.04	47.9	47.6	45.1
		Score test	2.46	2.01	1.10	95.3	93.8	89.0
3	5	Any	7.67	2.72	2.24	99.6	98.6	98.2
		S 1	2.52	0.84	0.73	0.1	0.1	0.1
		S 2	2.45	0.86	0.70	83.6	75.2	72.3
		S 3	2.78	1.01	0.84	99.4	98.2	97.7
		Score test	2.31	0.87	0.70	76.7	67.6	64.7
	6	Any	7.06	2.27	1.80	99.6	98.4	97.9
		cloglogS 1	2.41	0.77	0.63	0.1	0.1	0.1
		cloglogS 2	2.39	0.78	0.62	83.2	74.6	71.6
		cloglogS 3	2.21	0.62	0.49	99.3	98.0	97.4
		Score test	2.31	0.85	0.69	76.7	67.5	64.6
	7	Any	2.9	2.28	1.01	76.7	72.4	60.7
		avgHR	2.43	2.05	0.98	56.9	53.8	45.7
		RMST	2.37	2.02	0.98	45.8	45.2	42.1
		Score test	2.31	1.96	1.00	76.7	72.4	60.7

T1E: type 1 error rate; Pow: power; unadj: unadjusted; adj: adjusted; Holm: Bonferroni–Holm adj; HR: hazard ratio; avgHR: average HR; RMST: restricted mean survival time; S 1: scenario 1; S 2: scenario 2; S 3: scenario 3; cloglogS 1: complementary log–log scenario 1; cloglogS 2: complementary log–log scenario 2; cloglogS 3: complementary log–log scenario 3.

**Table 4.** T1E and Pow for unadj tests, multiplicity adj through the multivariate normal-based closed test (adj) and Holm tests, observed in 50,000 simulation runs for scenarios 4–6. Rows labelled ‘Any’ refer to the probability of rejecting the null hypothesis for at least one included parameter (i.e. family-wise T1E or Pow). Further rows show the rejection probability for each specific parameter included in the parameter set. The sample size in the simulation was 200 subjects per group. Values are presented as per cent.

Scenario	Set	Parameter	T1E unadj	T1E adj	T1E Holm	Pow unadj	Pow adj	Pow Holm
4	5	Any	6.41	2.46	1.74	94.6	88.4	85.2
		S 1	2.39	0.94	0.73	66.8	62.6	61.3
		S 2	2.61	1.05	0.73	83.5	77.1	74.9
		S 3	2.56	1.01	0.76	83.0	77.0	75.3
		Score test	2.45	0.99	0.70	91.6	85.0	81.7
	6	Any	6.24	2.31	1.60	94.5	88.1	84.8
		cloglogS 1	2.31	0.88	0.66	66.1	61.7	60.4
		cloglogS 2	2.55	0.99	0.69	83.2	76.5	74.1
		cloglogS 3	2.47	0.93	0.69	82.7	76.5	74.7
		Score test	2.45	0.98	0.69	91.6	84.9	81.6
	7	Any	3.27	2.40	1.12	92.8	90.9	84.7
		avgHR	2.47	1.98	1.04	90.6	89.3	83.8
		RMST	2.50	2.00	1.04	90.0	88.9	83.9
		Score test	2.45	1.97	1.07	91.6	90.2	84.5
5	5	Any	6.30	2.58	1.79	83.9	71.7	65.9
		S 1	2.58	1.10	0.80	63.6	54.9	51.9
		S 2	2.49	1.08	0.76	64.6	55.3	51.3
		S 3	2.60	1.11	0.79	54.0	46.9	44.2
		Score test	2.45	1.07	0.74	76.7	65.3	59.7
	6	Any	6.06	2.37	1.63	83.6	71.1	65.2
		cloglogS 1	2.51	1.04	0.75	63.1	54.2	51.1
		cloglogS 2	2.46	1.04	0.72	64.3	54.7	50.7
		cloglogS 3	2.39	0.93	0.66	53.5	46.1	43.4
		Score test	2.45	1.07	0.73	76.7	65.3	59.5
	7	Any	3.53	2.40	1.26	79.9	75.1	65.2
		avgHR	2.49	1.88	1.12	71.6	69.6	63.3
		RMST	2.49	1.88	1.03	77.0	73.4	64.1
		Score test	2.45	1.85	1.01	76.7	73.0	64.1
6	5	Any	7.16	2.58	1.97	98.1	94.6	93.2
		S 1	2.42	0.84	0.68	90.4	85.4	84.5
		S 2	2.63	0.95	0.73	88.0	82.2	81.1
		S 3	2.67	1.03	0.78	56.8	54.1	53.6
		Score test	2.51	0.93	0.73	96.7	92.7	91.0
	6	Any	6.58	2.17	1.65	98.1	94.4	93.0
		cloglogS 1	2.35	0.77	0.64	90.1	84.8	83.8
		cloglogS 2	2.57	0.89	0.68	87.7	81.7	80.6
		cloglogS 3	2.11	0.65	0.48	56.4	53.4	52.9
		Score test	2.51	0.92	0.72	96.7	92.6	91.0
	7	Any	3.36	2.44	1.16	98.0	97.3	94.7
		avgHR	2.52	2.03	1.10	97.5	97.0	94.6
		RMST	2.54	2.01	1.04	97.4	96.8	94.3
		Score test	2.51	1.97	1.05	96.7	96.3	94.3

T1E: type 1 error rate; Pow: power; unadj: unadjusted; adj: adjusted; Holm: Bonferroni–Holm adj; HR: hazard ratio; avgHR: average HR; RMST: restricted mean survival time; S 1: scenario 1; S 2: scenario 2; S 3: scenario 3; cloglogS 1: complementary log–log scenario 1; cloglogS 2: complementary log–log scenario 2; cloglogS 3: complementary log–log scenario 3.

Without adjustment, the type I error rate for the global null hypothesis of no difference in any included parameter was in the order of 6.5% to 7.5% in the studied scenarios. Multiplicity adjustment using the proposed multivariate normal approximation resulted in type I error rates close to the nominal 2.5%. Some inflation was still observed for parameter sets containing untransformed survival probabilities, which likely results from the inaccuracy even of the univariate approximation for these parameters (see column ‘T1E adj’ in Tables 3 and 4). Adjustment using the Bonferroni–Holm test resulted in strictly conservative tests and adjusted significance levels below those of the multivariate normal-based closed test (see column ‘T1E adj’ and ‘T1E Holm’ in the result tables).

The power of the multivariate normal-adjusted tests for the global null hypothesis was on average 4.0 percentage points below the power of corresponding unadjusted tests. For comparison, Bonferroni-adjusted tests were on average 7.7 percentage points less powerful than the unadjusted tests. (See columns regarding power and rows with parameter label ’Any’ in Tables 3 and 4.) Similarly, the power for elementary hypothesis tests was on average 4.4 percentage points lower with the multivariate normal-based closed test compared to unadjusted univariate tests. The Bonferroni–Holm procedure resulted on average in 7.2 percentage points lower power compared to unadjusted tests.

That means, averaged across the studied settings, the proposed testing procedure retains almost half the power loss, which would occur with a simpler Bonferroni–Holm approach.

When comparing the approach to test multiple parameters with a single Cox model score test, in scenarios with strong non-proportionality of hazard functions (scenarios 1, 2 and 3), the hypothesis test for a difference in 3-year survival or 3-year cloglog-transformed survival was of similar (scenario 1) or larger power (scenarios 2 and 3) compared to the score test.

When including the difference for 1-year, 2-year, and 3-year survival (or cloglog-transformed survival) and the score test in one parameter set and adjusting for multiple testing, the power to show a difference in at least one considered parameter was similar (Scenario 1) or considerably larger (Scenarios 2 and 3) than the power of a single unadjusted score test. Further, the power to show a difference in at least one parameter under multiplicity adjustment was similar to the unadjusted power of the most powerful univariate comparison (either 3-year survival or score test).

This implies that, first, under severely non-proportional hazards, testing for differences at a well-chosen milestone time-point can be more powerful than the score test. And, secondly, testing several milestone time points and adjusting for multiplicity will often be a better choice than selecting one time-point in advance and avoid multiple testing, as the multiplicity adjustment with the proposed method will mostly maintain the power of the most powerful univariate comparison.

When comparing the score test for the Cox model hazard ratio with tests for the two other summary measures (average hazard ratio and RMST difference), the score test performs by far best in the three scenarios with strongly non-proportional hazards.

In scenarios with proportional hazards (scenario 4) or moderate non-proportionality (scenarios 5 and 6), the score test was more powerful, in the order of 10 percentage points, than the best comparison for survival at either milestone time point. Also, the unadjusted power of the score test was larger than the adjusted power to reject at least one of the considered null hypotheses by two to five percentage points. Tests for average hazard ratio and RMST difference had almost identical power as the score test in scenarios 4 and 6 and when combining all three tests, the adjusted power to reject at least one null hypothesis was also at the almost same value as the power of the unadjusted tests. In scenario 6, the score test and the test for RMST difference both had a power of ∼ 77%, whereas the test for average hazard ratio was at 71.6%. Still, the power of the better tests was almost retained in adjusted power to reject at least one null hypothesis with a value of 75%.

Similar patterns were observed with sample sizes of 50 per group, see Tables S1 and S2 in the Supplemental material. As the main differences to the moderate sample size setting, tests based on cloglog survival probabilities were increasingly conservative in the small sample setting with family-wise type I error rates between 1.65% and 2.47% when using the multivariate normal adjustment. Similar to the larger sample size, tests that included untransformed survival probabilities were liberal, with family-wise type I error rates up to 2.97%. The family-wise type I error rate was controlled at the 2.5% level for the parameter set comprising the average hazard ratio, RMST difference and the score test.

Taken together, the simulation results show that in terms of power to show at least some difference, the score test (or logrank test) is in most settings a robust choice. However, to increase robustness or to aid in the interpretation of the pattern of differences between survival curves, the test may be complemented by tests for further parameters and, when applying the proposed multiplicity adjustment, the adjusted overall power to find some difference will typically remain at a very similar level as the power of the best-included test.

## 5. Data example

As an illustrating example, we considered comparisons from the study ‘Pembrolizumab alone or with chemotherapy versus cetuximab with chemotherapy for recurrent or metastatic squamous cell carcinoma of the head and neck (KEYNOTE-048): a randomised, open-label, phase 3 study’ by Burtness et al.^22^ The survival curves shown in this study exhibit obvious properties of non-proportional hazards. For the comparison of pembrolizumab alone versus cetuximab with chemotherapy, survival curves were crossing, with better survival under cetuximab with chemotherapy in the first eight months and subsequent better survival under pembrolizumab. In the comparison of pembrolizumab with chemotherapy versus cetuximab with chemotherapy, survival curves were almost equal in both groups for the first eight months with subsequent separation of the two curves, showing a benefit for the pembrolizumab group.

**Figure 4.**
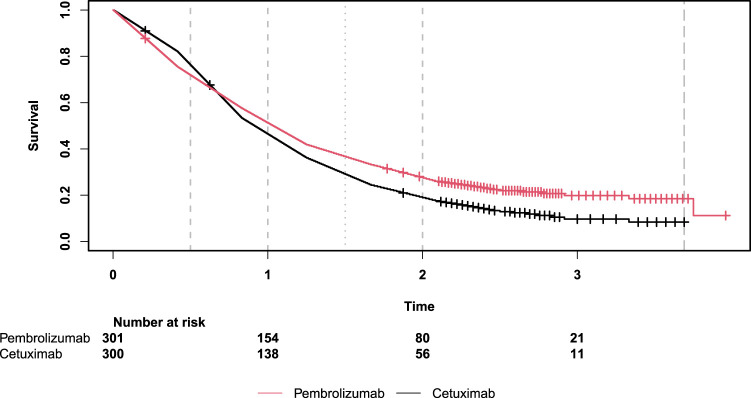
Estimated survival functions for the example data based on Figure 2D of Burtness et al.^22^

We reconstructed a data set for the comparison of overall survival in the full population under pembrolizumab alone versus cetuximab with chemotherapy based on the numbers at risk and the number of censored event times, which are given for every five-month interval in Figure 2D of this publication.^22^ In the reconstructed data set, event times and censoring times, respectively, were equally spread within each five-month interval. The survival functions for the reconstructed data set were estimated using the Nelson–Aalen method and are shown in Figure 4. The data set contains 301 subjects with 237 events in the pembrolizumab alone group and 300 subjects with 264 events in the cetuximab with chemotherapy group. The overall median and maximum follow-up times are 0.96 years and 3.96 years. In the study by Burtness et al.,^22^ confirmatory tests were performed for the Cox model hazard ratio between groups. As discussed, under non-proportional hazards, the expected value of the Cox model hazard ratio depends on trial characteristics such as the length of recruitment and follow-up periods and other parameters may be more appropriate to quantify the treatment effect, in particular, under crossing survival curves (see, e.g. Magirr and Burman^9^). Accordingly, survival functions were further characterized in Burtness et al.^22^ by reporting 0.5-year survival, 1-year survival and median survival times, however, no inference for the between-group difference of these parameters and, consequently, no adjustment for simultaneous inference was provided. In our example, assume there is some information from previous studies that the survival curves will likely show late separation and a standard analysis may hence be affected by non-proportional hazards.

We consider two alternative analysis approaches. The first approach is focused on establishing a difference between survival curves based on a small set of parameters, the second one aims at a characterization of differences by confidence intervals for a larger set of parameters.

In the first approach, two primary null hypotheses for the overall differences in survival functions and for the difference in 2-year survival probabilities are defined and tested, respectively, with the Cox model score test and the Wald test for survival differences. Multiplicity adjustment at the simultaneous one-sided significance level of 0.025 is applied, using the closed test based on the multivariate normal distribution with asymptotic covariance estimate. This analysis is intended to show at least some benefits of pembrolizumab (Group 1) over cetuximab (Group 0). Two tests are combined to complement a possible lack in power of the score test compared to the potentially large difference in survival at a late milestone time point. The according null hypotheses are $H_{01}:\lambda_{1}(s)\geq \lambda_{0}(s),\forall s\leq 0\leq 3.5$ and $H_{02}:S_{1}(2)\leq S_{0}(2)$ .

The resulting unadjusted one-sided p -values for $H_{01}$ and $H_{02}$ are $p_{1}=0.0100$ and $p_{2}=0.0053$ . The according multiplicity-adjusted one-sided p -values, resulting from the multivariate normal-based closed test, are $p_{1,\mathrm{adj}}=0.0100$ and $p_{2,\mathrm{adj}}=0.0082$ . Hence, both null hypotheses are rejected at the one-sided family-wise significance level of 0.025. Furthermore, the adjustment did not change the p -value of the score test, and only by a small amount increased the p -value of the test for 2-year survival differences. In this example, the estimated correlation between the two test statistics was 0.87, and this large correlation entails a very modest adjustment to the p -values.

To further explore the power of the considered analysis approach in the setting of the example, we performed a bootstrap simulation study. Bootstrap samples of the same size as the original data were obtained by sampling patients with replacement from the example data set. The above analyses were applied to 50,000 bootstrap samples at the one-sided 0.025 significance level. For the score test, the empirical power values without multiplicity adjustment, with adjustment using the multivariate normal-based closed test and with Bonferroni–Holm adjustment were 65.8%, 63.2% and 60.9%. The respective power values for the 2-year survival difference were larger with 72.0%, 68.2% and 64.8%.

In the second example analysis approach, the difference in survival curves is characterized by a parameter set that includes the differences in 0.5-year survival, 1-year survival, 2-year survival, median survival times and 3.5-year RMST. Simultaneous two-sided 95% confidence intervals for these parameters are calculated using the multivariate normal adjustment with the asymptotic covariance estimate.

The results of the second analysis are shown in Table 5. In this example, the width of Bonferroni-adjusted confidence intervals is 1.064 times the width of multivariate normal-adjusted intervals.

**Table 5.** Analysis of example data based on Figure 2D of Burtness et al.^22^ Selected parameters of the survival function under pembrolizumab and cetuximab are compared using unadj confidence intervals, simultaneous confidence intervals with adjustment based on the MVN distribution (MVN adjusted) and Bonferroni-adjusted intervals.

				95% Confidence intervals		
Parameter	Pembro.	Cetux.	Difference	Unadjusted	MVN adjusted	Bonferroni
0.5-year survival	0.721	0.763	−0.042	[−0.112, 0.028]	[−0.129, 0.044]	[−0.134, 0.049]
1-year survival	0.514	0.465	0.049	[−0.030, 0.129]	[−0.049, 0.148]	[−0.056, 0.155]
2-year survival	0.277	0.189	0.088	[0.021, 0.155]	[0.005, 0.171]	[−0.001, 0.177]
Median survival	1.037	0.915	0.122	[−0.075, 0.319]	[−0.121, 0.365]	[−0.136, 0.381]
3.5-year RMST	1.436	1.232	0.204	[0.027, 0.381]	[−0.015, 0.422]	[−0.029, 0.437]

unadj: unadjusted; MVN: multivariate normal; RMST: restricted mean survival time.

The analysis shows that regarding 0.5-year survival, lower efficacy of pembrolizumab alone or with chemotherapy versus cetuximab cannot be ruled out, with a difference up to 13 percentage points at the adjusted 95% confidence level. At 1-year, the relation has reversed with point estimates for survival probabilities and for the median, which is close to 1 year in both groups, favouring pembrolizumab. Albeit uncertainty remains at this time point, reflected in the confidence intervals that cover the possibility of no between-groups difference in 1-year survival and in the median survival times. Only at longer time spans point estimates and confidence intervals for 2-year survival and the 3.5-year RMST difference support the conclusion of larger benefit under pembrolizumab.

## 6. Software implementation

The proposed methods were implemented in the R^42^ function nphparams(). This function was added to the previously published R library nph,^5^ which provides functions to simulate and analyse survival data under non-proportional hazards. The function makes use of the R library multcomp,^39^ which provides general functions for simultaneous inference with multivariate normal statistics. The example data set of Section 5, too, was added to the nph package under the name pembro. Appendix A.1 contains R code that may be applied to reproduce the exemplary data analysis.

Computing time is not restrictive for the proposed methods. In the analysis of the example data, the application of the nphparams() R-function to parameter set 2 (comprising 0.5-, 1- and 2-year survival difference, median survival difference and RMST) takes 0.3 s when using the asymptotic covariance estimate and 0.4, 0.5 or 2.7 s when using the perturbation approach with 500 (default), 1000 or 10,000 perturbations on a 3.4 GHz processor.

## 7. Discussion

In the absence of the proportional hazard assumption, the exact definition of a survival benefit under treatment versus control is ambiguous. Essentially, two distribution functions need to be compared and different aspects of these distributions may receive different emphases depending on personal preferences or circumstances. For example, a survey by Shafrin et al.^43^ among melanoma patients and lung cancer patients and their treating physicians found that patients on average preferred a larger chance for increased long-term survival and in exchange were more willing to accept increased short-time risk for mortality as opposed to their physicians.

Consequently, to formally establish a benefit of treatment over control in a clinical trial under non-proportional hazards, more than one parameter for the difference in survival functions needs to be regarded. Our aim was to provide a formal inference framework that is applicable, both, under non-proportional and proportional hazard settings, that includes a wide range of suitable parameters and that allows for an efficient parametric multiplicity adjustment to control the type I error rate of hypothesis tests and the simultaneous coverage of confidence intervals.

All considered parameter estimates essentially are a function of the observed event process and as such can be combined in a joint counting process framework that establishes their asymptotic multivariate normal distribution. Simultaneous inference based on this distributional approximation results in more powerful procedures than adjustments such as the Bonferroni–Holm method, which does not take into account the underlying distribution. In particular when parameters are highly correlated, as is, for example, the case for combinations such as 3-year survival and RMST in our simulation scenarios, only moderate multiplicity adjustment is required and the proposed methods result in little loss in efficacy compared to unadjusted univariate analyses.

Which parameters to include in an actual analysis may depend on clinical reasoning and statistical considerations. A set of x-year survival comparisons may be useful to provide a concise characterization of differences between two survival curves. With small sample sizes, these comparisons should be made using the cloglog transformation to improve the accuracy of the asymptotic-based inference. Summary measures such as RMST difference or average hazard ratio may be added to provide an overall estimate of the treatment effect across the considered time interval. The score test may be included to maintain robust power for hypothesis testing purposes, with further interpretation supported by a small set of well-interpretable parameters. The number of considered parameters could be larger than in the simulation study, however, more parameters would result in more conservative multiplicity adjustment (depending on their correlations) while the gain in information from an increasingly large number of parameters may be limited.

Of note, in the absence of proportional hazards, the Cox model hazard ratio is not robust to design characteristics as it depends on the timing of events and hence can be affected, for example, by the recruitment rate and length of follow-up. Thus the traditional hazard ratio is of limited use to quantify differences in survival curves under non-proportional hazards. In particular with crossing survival curves (or more generally crossing hazard curves) the hazard ratio estimate should be interpreted with care. The logrank test, or equivalently the Cox model score test, however, is calculated under a global null hypothesis of equal hazard functions and therefore is a viable approach to test the null hypothesis of equal survival functions. Rejection of this null hypothesis implies that there is at least some difference between two survival functions, however, it does not provide an interpretation of how strong this difference is and it does not rule out effects being of different sizes or even show into opposite directions at different time points.

As an alternative to the proposed multidimensional parametric approach, conclusions could also be drawn from overall inspection of the observed survival functions, and simultaneous inference could be based on confidence bands with simultaneous coverage.^38,44^ This would correspond to a fully non-parametric approach. Simultaneous confidence bands are typically considerably wider than simultaneous confidence intervals calculated specifically for a small set of predefined time points. For illustration, an application of this method to the example data of Section 5 and a comparison to methods proposed in this paper can be found in the Supplemental material. Such an approach may be suitable to inform the treatment decision of an individual patient, however, it is not suitable to define success criteria regarding efficacy in a clinical trial or when evaluating treatment strategies in clinical practice. To interpret and communicate the effects of drugs with a complex pattern of efficacy over time, a set of quantifying parameters seems to be a good compromise between a single parameter, such as the hazard ratio, and a completely non-parametric approach of regarding the overall survival curves.

The multiple testing procedures described in Section 3 may be extended towards more complex methods. A serial gate-keeping procedure^45^ may be used to first show a difference between treatment and control by testing an intersection hypothesis for a small set of parameters for which a high power is expected, and in case of success assess the survival differences in detail with respect to a larger set of relevant parameters. For example, in the example of Section 5, the intersection hypothesis test comprising the logrank test and the test for 2-year survival could be used to establish some difference and act as gate-keeper for the subsequent analysis of the larger parameter set. Of note, the rejection of the gate-keeping intersection hypothesis in the first step does not automatically imply the rejection of the corresponding elementary null hypotheses of the first set, because these are not included in the closed testing procedure that corresponds to the gate-keeping approach. To define more complex testing procedures with several levels, hierarchies or different weights for the included tests, parametric graphical multiple testing procedures^46–48^ could be defined using the estimated covariance matrix.

The first step in our example is similar in spirit to the combined test proposed by Royston and Parmar.^49^ They suggested to perform a MaxCombo test that includes the logrank test and RMST differences at several time points. Royston and Parmar use permutation (or an approximation thereof) to calculate p -values. However, their test can as well be performed within the asymptotic multivariate normal framework we presented, and may be supplemented by simultaneous confidence intervals for the included RMST differences.

Though not covered in the present work, the simultaneous inference framework may be extended to include stratified analyses. One way to do so would be to estimate the parameters of interest and their covariance matrix for each stratum separately and then calculate a weighted average of the per-stratum estimates and the corresponding covariance matrix. Weights could correspond to stratum size, however, further investigations into the ideal choice of weights may be warranted.

Also, weighted logrank statistics may be included in the inference framework as a further extension. One could also consider performing interim analyses to allow for early stopping^14,50^ and adaptations such as sample size reassessment^51^ and modification of the set of parameters, for example, adding milestone analyses at later time points. Depending on which type of data is considered for the adaptations,^51,52^ appropriate adaptive tests have to be implemented.

In summary, simultaneous inference for a predefined set of survival parameters allows for a robust assessment of treatment efficacy under non-proportional hazards. The required multiplicity adjustments can be performed efficiently based on their asymptotic joint normal distribution.

## Supplemental Material

sj-pdf-1-smm-10.1177_09622802241231497 - Supplemental material for Simultaneous inference procedures for the comparison of multiple characteristics of two survival functions Supplemental material, sj-pdf-1-smm-10.1177_09622802241231497 for Simultaneous inference procedures for the comparison of multiple characteristics of two survival functions by Robin Ristl, Heiko Götte, Armin Schüler, Martin Posch and Franz König in Statistical Methods in Medical Research
