# Supplementary material for: Simultaneous inference procedures for the comparison of multiple characteristics of two survival functions
Source: Stat Methods Med Res. 2024 Mar 11;33(4):589–610. doi: 10.1177/09622802241231497 (PMC11025310; doi:10.1177/09622802241231497)
Supplement: sj-pdf-1-smm-10.1177_09622802241231497 - Supplemental material for Simultaneous inference procedures for the comparison of multiple characteristics of two survival functions [file sj-pdf-1-smm-10.1177_09622802241231497.pdf]

Supplemental material to "Simultaneous inference procedures for the comparison of multiple characteristics of two survival functions"

1 Simulation results for the perturbation approach

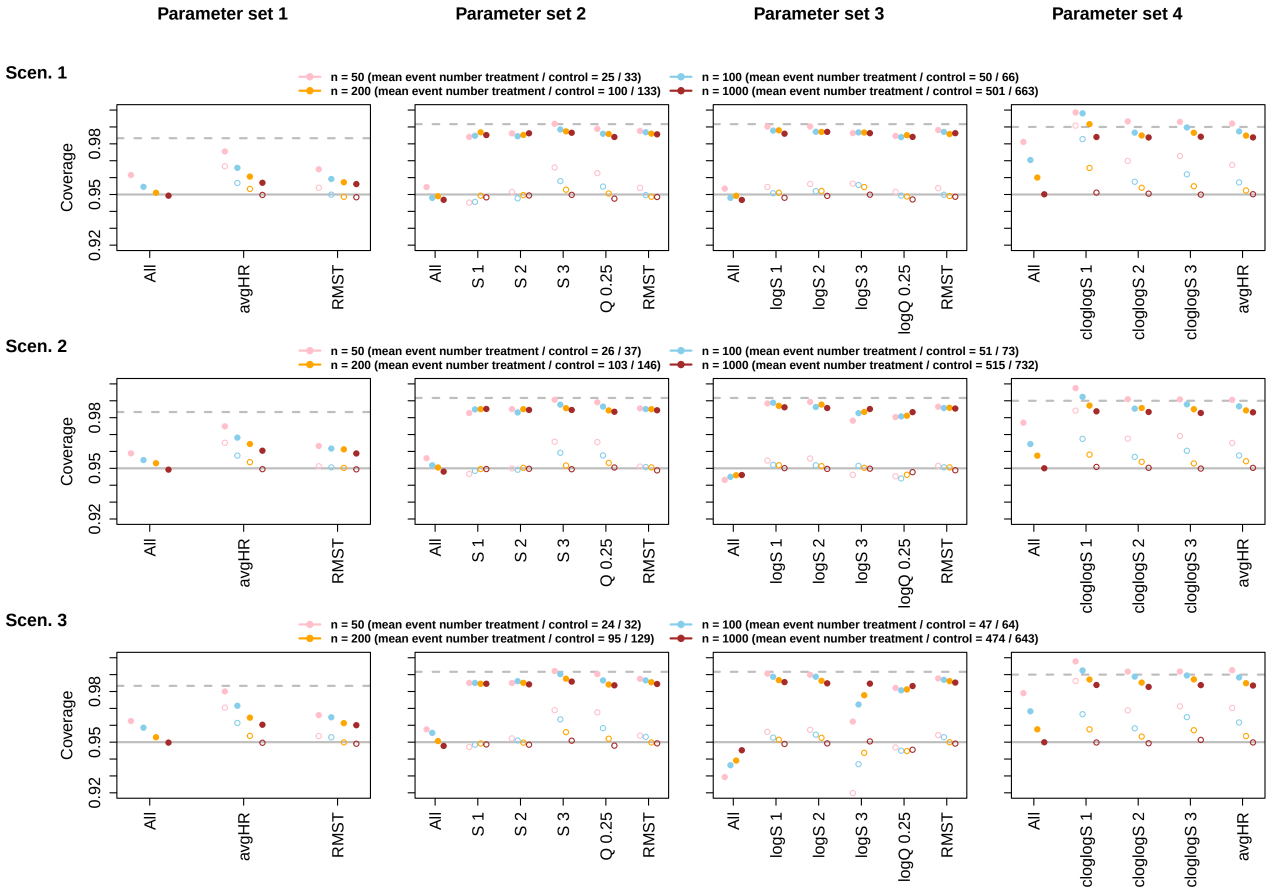

Figure S1: Empirical coverage of confidence intervals for Scenarios 1-3, based on the multivariate normal distribution with perturbation covariance matrix estimate. Filled circles show the simultaneous coverage (All) and univariate coverage probability of multiplicity adjusted intervals for single parameters (abbreviations as in Table 2 of the main manuscript). Open circles represent the coverage of unadjusted univariate confidence intervals. Error bars represent 95% Wald confidence intervals for the respective coverage probabilities. For comparison, the horizontal solid line indicates the nominal coverage of 95%, and the horizontal dashed line indicates the univariate confidence level that would result from a Bonferroni adjustment for the respective number of parameters.

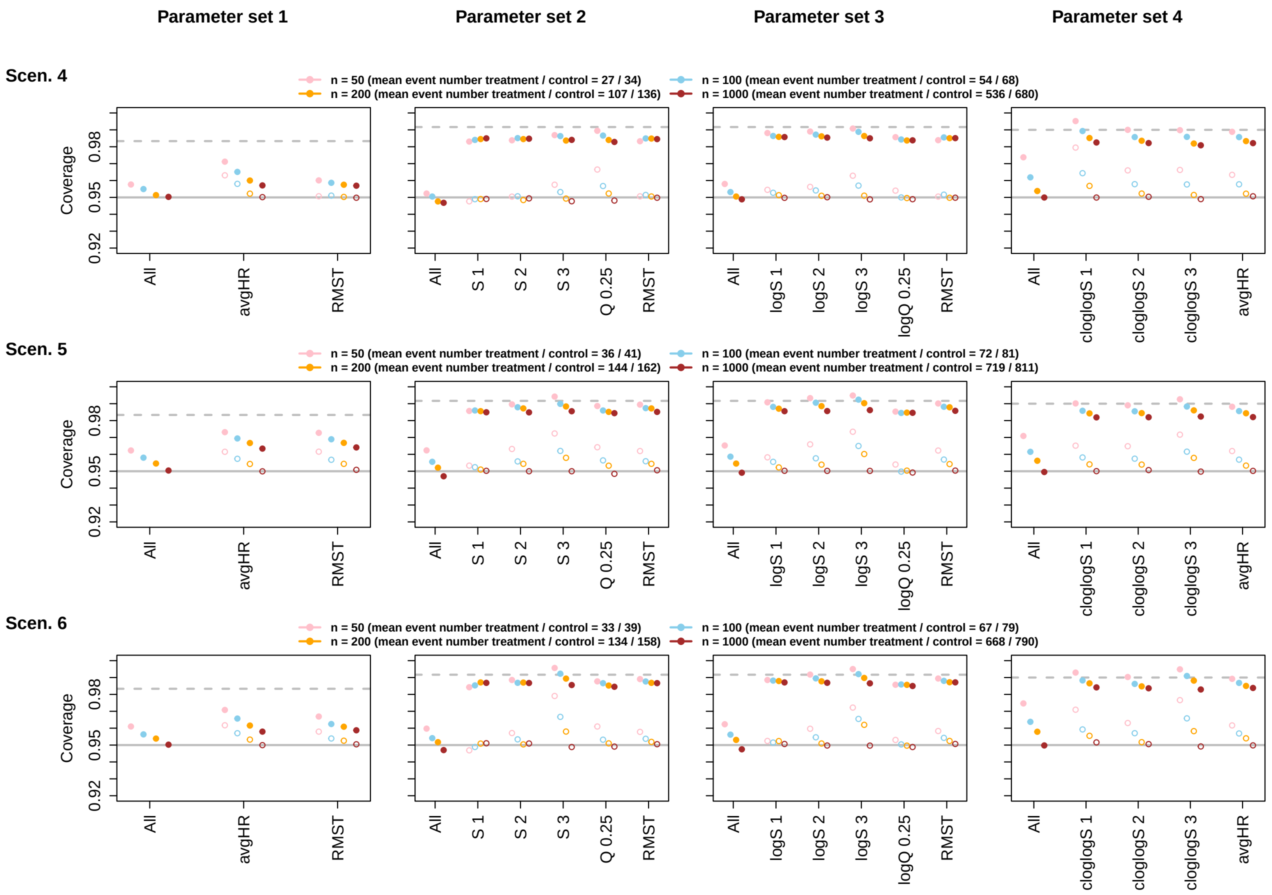

Figure S2: Empirical coverage of confidence intervals for Scenarios 4-6, based on the multivariate normal distribution with perturbation covariance matrix estimate. Further details as in Figure S1.

2 Simulation results for parameter sets that include the Cox model hazard ratio

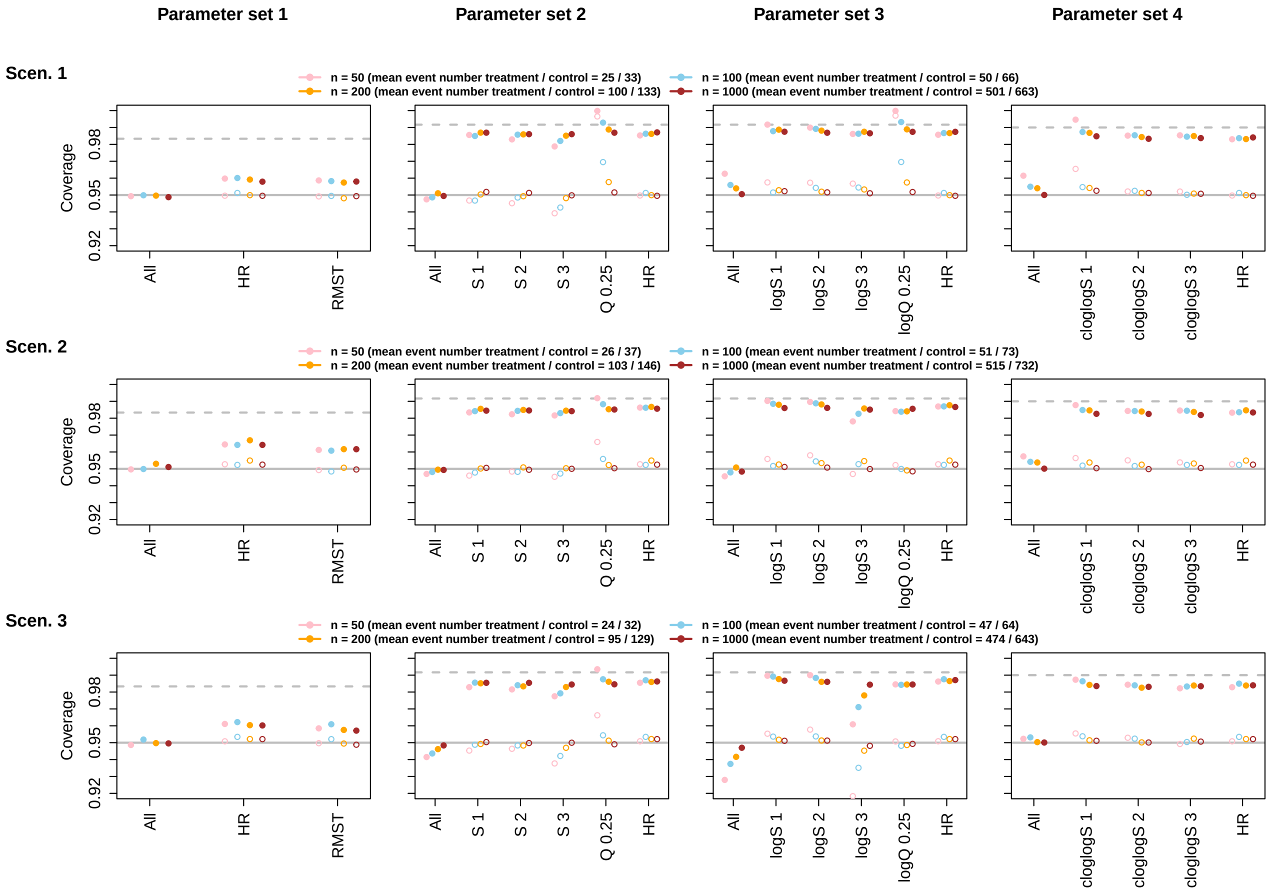

Figure S3: Empirical coverage of confidence intervals for parameter sets including the Cox model hazard ratio for Scenarios 1-3. Intervals are based on the multivariate normal distribution with asymptotic covariance matrix estimate. In case of the hazard ratio, coverage was calculated for the expected value of the hazard ratio under the specific scenario assumptions. With sample size 50 per group under scenario 1, in 33 out of 50,000 runs the variance of the 1-year survival probability was not estimable due to zero events within one year in one group. These runs were excluded from the calculations. Further details as in Figure S1.

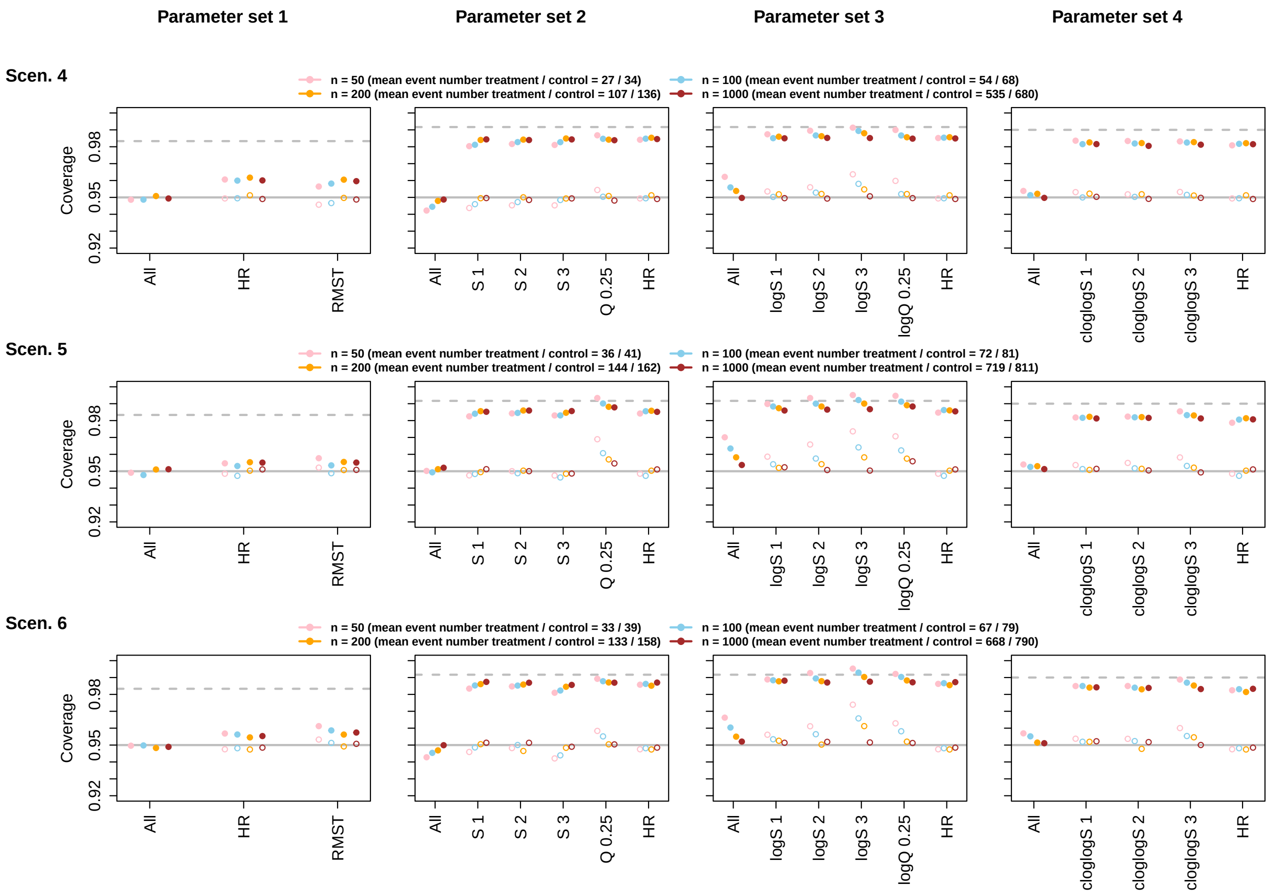

Figure S4: Empirical coverage of confidence intervals for parameter sets including the Cox model hazard ratio for Scenarios 4-6. Intervals are based on the multivariate normal distribution with asymptotic covariance matrix estimate. In case of the hazard ratio, coverage was calculated for the expected value of the hazard ratio under the specific scenario assumptions. Further details as in Figure S1.

3 Simulation results for parameter sets 5 to 7 with $n = 50$ per group

Table S1: Type I error rate (T1E) and power (Pow) for unadjusted (unadj) tests, multiplicity adjustment through the multivariate-normal based closed test (adj) and Bonferroni-Holm adjusted (Holm) tests, observed in 50,000 simulation runs for scenarios 1-3. Rows labelled 'Any' refer to the probability to reject the null hypothesis for at least one included parameter (i.e. family-wise type I error rate or power). Further rows show the rejection probability for each specific parameter included in the parameter set. The sample size in the simulation was 50 subjects per group. In 32 out of 50,000 runs under Scenario 1, the variance of the 1-year survival probability was not estimable due to zero events within one year in one group. These runs were excluded from the calculations. Values are presented as percent.

Scenario	Set	Parameter	T1E unadj	T1E adj	T1E Holm	Pow unadj	Pow adj	Pow Holm
1	5	Any	8.05	2.97	2.38	57.0	38.4	34.8
		S 1	2.79	1.02	0.84	2.8	1.7	1.6
		S 2	2.80	1.06	0.87	33.1	21.4	19.6
		S 3	2.71	1.01	0.79	41.7	28.5	26.3
		Score test	2.38	0.77	0.60	38.2	23.3	20.8
	6	Any	6.39	1.69	1.26	54.2	33.5	29.7
		cloglogS 1	1.85	0.32	0.22	1.9	0.9	0.9
		cloglogS 2	2.47	0.80	0.64	30.9	18.6	16.9
		cloglogS 3	1.98	0.51	0.38	38.6	23.7	21.4
		Score test	2.38	0.72	0.55	38.2	22.7	20.1
	7	Any	3.26	2.29	1.08	39.5	33.0	22.8
		avgHR	2.58	2.01	1.02	31.3	28.6	21.4
		RMST	2.54	1.96	1.03	30.7	28.5	21.3
		Score test	2.38	1.82	1.02	38.3	32.3	22.6
2	5	Any	7.68	2.63	2.06	82.4	70.1	65.9
		S 1	2.90	0.94	0.74	0.5	0.5	0.5
		S 2	2.77	1.02	0.86	33.7	24.2	21.8
		S 3	2.49	0.85	0.68	81.0	68.8	64.8
		Score test	2.36	0.78	0.63	40.9	27.8	24.8
	6	Any	6.52	1.84	1.42	80.8	65.8	60.9
		cloglogS 1	2.27	0.55	0.43	0.4	0.4	0.4
		cloglogS 2	2.46	0.80	0.64	31.8	21.6	19.4
		cloglogS 3	1.96	0.51	0.41	79.3	64.7	60.0
		Score test	2.36	0.76	0.59	40.9	27.5	24.4
	7	Any	3.37	2.30	1.19	41.0	33.5	23.3
		avgHR	2.66	1.99	1.11	19.5	17.8	13.1
		RMST	2.58	2.00	1.10	15.9	15.3	12.3
		Score test	2.36	1.77	1.09	40.9	33.5	23.3
3	5	Any	7.94	2.92	2.36	69.4	54.2	50.1
		S 1	2.72	1.04	0.85	0.5	0.5	0.4
		S 2	2.68	0.92	0.76	32.1	21.5	19.0
		S 3	2.79	1.07	0.87	63.6	49.9	46.4
		Score test	2.28	0.71	0.56	23.7	14.9	13.1
	6	Any	6.46	1.82	1.38	65.7	46.9	42.0
		cloglogS 1	2.17	0.58	0.45	0.4	0.4	0.3
		cloglogS 2	2.29	0.68	0.55	30.2	19.0	16.4
		cloglogS 3	1.88	0.51	0.39	59.7	42.7	38.5
		Score test	2.28	0.68	0.52	23.7	14.6	12.7
	7	Any	3.08	2.16	1.08	24.4	19.0	12.0
		avgHR	2.52	1.93	1.03	17.6	15.1	10.6
		RMST	2.34	1.84	1.02	13.6	12.8	9.7
		Score test	2.28	1.77	1.00	23.7	18.7	12.0

Table S2: Type I error rate (T1E) and power (Pow) for unadjusted (unadj) tests, multiplicity adjustment through the multivariate-normal based closed test (adj) and Bonferroni-Holm adjusted (Holm) tests, observed in 50,000 simulation runs for scenarios 4-6. Rows labelled 'Any' refer to the probability to reject the null hypothesis for at least one included parameter (i.e. family-wise type I error rate or power). Further rows show the rejection probability for each specific parameter included in the parameter set. The sample size in the simulation was 50 subjects per group. Values are presented as percent.

Scenario	Set	Parameter	T1E unadj	T1E adj	T1E Holm	Pow unadj	Pow adj	Pow Holm
4	5	Any	6.88	2.80	2.05	51.7	35.0	29.8
		S 1	2.77	1.18	0.87	23.6	16.7	14.9
		S 2	2.72	1.21	0.93	32.0	22.6	19.8
		S 3	2.77	1.15	0.88	31.8	22.4	19.8
		Score test	2.36	0.94	0.68	37.1	24.5	20.6
	6	Any	6.10	2.17	1.47	49.5	31.4	26.1
		cloglogS 1	2.40	0.83	0.58	21.2	13.9	12.2
		cloglogS 2	2.44	1.00	0.72	30.0	20.1	17.2
		cloglogS 3	2.38	0.85	0.62	29.8	19.8	17.3
		Score test	2.36	0.90	0.64	37.1	23.8	19.8
	7	Any	3.32	2.33	1.19	41.9	35.8	25.8
		avgHR	2.60	2.02	1.15	36.9	33.1	24.8
		RMST	2.72	2.07	1.15	37.4	33.8	25.3
		Score test	2.36	1.86	1.11	37.1	33.0	25.0
5	5	Any	5.97	2.32	1.59	38.6	24.2	19.5
		S 1	2.50	1.11	0.79	21.6	14.3	12.0
		S 2	2.35	0.91	0.62	21.8	14.4	11.8
		S 3	2.27	0.89	0.64	18.2	12.2	10.0
		Score test	2.22	0.84	0.55	25.8	16.1	12.8
	6	Any	5.28	1.90	1.17	36.9	22.0	17.1
		cloglogS 1	2.28	0.92	0.60	20.2	12.7	10.5
		cloglogS 2	2.13	0.78	0.50	20.8	13.3	10.5
		cloglogS 3	1.78	0.62	0.41	16.7	10.5	8.5
		Score test	2.22	0.83	0.52	25.8	15.9	12.4
	7	Any	3.29	2.01	1.12	30.7	24.5	17.4
		avgHR	2.35	1.66	1.04	24.2	21.1	16.2
		RMST	2.20	1.53	0.87	26.6	22.3	16.2
		Score test	2.22	1.48	0.86	25.8	21.4	15.9
6	5	Any	6.82	2.55	1.89	59.9	41.4	36.9
		S 1	2.69	1.06	0.84	37.9	26.6	24.4
		S 2	2.55	1.01	0.77	35.0	24.5	22.3
		S 3	2.32	0.77	0.54	19.4	13.5	12.4
		Score test	2.31	0.84	0.64	46.1	30.9	27.2
	6	Any	5.86	1.94	1.42	57.8	38.0	33.2
		cloglogS 1	2.36	0.83	0.64	35.6	23.2	21.0
		cloglogS 2	2.34	0.84	0.63	33.6	22.2	20.0
		cloglogS 3	1.65	0.42	0.31	17.4	11.2	10.2
		Score test	2.31	0.83	0.61	46.1	30.5	26.5
	7	Any	3.26	2.23	1.17	53.5	47.2	36.1
		avgHR	2.54	1.96	1.12	49.3	45.1	35.6
		RMST	2.35	1.80	0.99	48.7	44.6	35.0
		Score test	2.31	1.74	1.01	46.1	42.5	34.7

4 Data example simultaneous confidence bands

The aim of this supplementary section is to illustrate the use of confidence bands with simultaneous coverage for the difference between two survival functions in comparison to the methods proposed in the main manuscript. A 95% confidence band was calculated for the example data of Section 5 of the main manuscript using the perturbation method described by Parzen et al. [1]. The number of perturbation samples was set to 1000. The confidence intervals for the three specific time points 0.5, 1 and 2 years were extracted from the obtained bands. For comparison, simultaneous 95% confidence intervals for the survival difference at 0.5, 1 and 2 years were calculated using the multivariate normal approximation as described in Section ... of the main manuscript. The resulting confidence intervals are shown in Table S3. The intervals obtained from the simultaneous confidence bands are wider than the intervals that were adjusted specifically for the three selected time-points by a factor of 1.35 to 1.39. For further illustration, the point estimates, simultaneous confidence bands and the confidence intervals specifically adjusted to the three selected time-points are shown in Supplementary Figure S5.

Table S3: Additional analysis of the example data of Section 5 of the main manuscript. The difference in survival probabilities at 0.5, 1 and 2 years under pembrolizumab versus cetuximab is analysed using simultaneous confidence intervals with adjustment based on the multivariate normal distribution (MVN adjusted) and intervals obtained from simultaneous confidence bands, which provide simultaneous coverage across all observed event times. The width of both interval types is compared in the last column in terms of a width ratio of simultaneous confidence band-derived intervals versus MVN adjusted intervals for the three studied time-points.

Parameter	Pembro.	Cetux.	Difference	MVN adjusted	Simultaneous	Width ratio
Survival difference 0.5	0.721	0.763	-0.042	[-0.126, 0.041]	[-0.156, 0.071]	1.35
Survival difference 1	0.514	0.465	0.049	[-0.046, 0.145]	[-0.084, 0.183]	1.39
Survival difference 2	0.277	0.189	0.088	[0.007, 0.169]	[-0.022, 0.198]	1.36

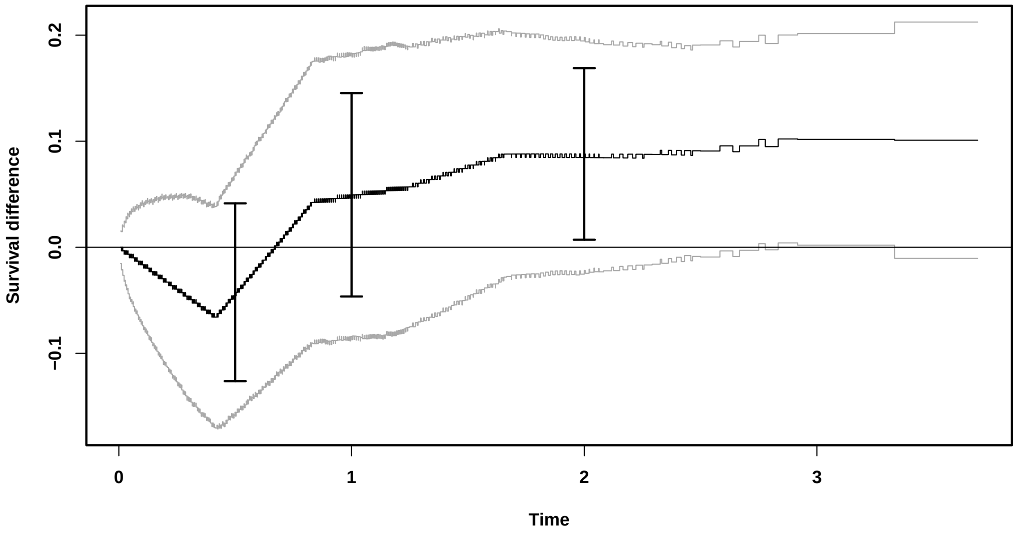

Figure S5: Estimated survival difference (black step function) and confidence bands with simultaneous 95% coverage (grey step functions representing lower and upper limit) calculated for the example data of Section 5 of the main manuscript. For comparison, simultaneous 95% confidence intervals specifically adjusted to three selected time points at 0.5, 1 and 2 years, using the multivariate normal approximation, are shown (vertical error bars).

References

[1] Parzen M, Wei L and Ying Z. Simultaneous confidence intervals for the difference of two survival functions. *Scandinavian Journal of Statistics* 1997; 24(3): 309–314.
